# Self-Scheduled LPV Control of Asymmetric Variable-Span Morphing UAV

**DOI:** 10.3390/s23063075

**Published:** 2023-03-13

**Authors:** Jihoon Lee, Seong-Hun Kim, Hanna Lee, Youdan Kim

**Affiliations:** 1 Department of Aerospace Engineering, Seoul National University, Seoul 08826, Republic of Korea; 2 Institute of Advanced Aerospace Technology, Seoul National University, Seoul 08826, Republic of Korea

**Keywords:** morphing aircraft, unmanned aerial vehicle, linear parameter-varying control, gain scheduling, robust control, flight control system, control augmentation system, autopilot, trajectory tracking, nonlinear guidance

## Abstract

In this study, a novel framework for the flight control of a morphing unmanned aerial vehicle (UAV) based on linear parameter-varying (LPV) methods is proposed. A high-fidelity nonlinear model and LPV model of an asymmetric variable-span morphing UAV were obtained using the NASA generic transport model. The left and right wing span variation ratios were decomposed into symmetric and asymmetric morphing parameters, which were then used as the scheduling parameter and the control input, respectively. LPV-based control augmentation systems were designed to track the normal acceleration, angle of sideslip, and roll rate commands. The span morphing strategy was investigated considering the effects of morphing on various factors to aid the intended maneuver. Autopilots were designed using LPV methods to track commands for airspeed, altitude, angle of sideslip, and roll angle. A nonlinear guidance law was coupled with the autopilots for three-dimensional trajectory tracking. A numerical simulation was performed to demonstrate the effectiveness of the proposed scheme.

## 1. Introduction

An advanced aerial platform known as a morphing unmanned aerial vehicle (UAV) is capable of controlled, large-scale shape transformations while in flight, which improves efficiency, adaptability, and performance across a range of flight scenarios and missions. Modern UAV missions are becoming increasingly complex [1,2], making it difficult for fixed-shape aircraft to perform their assigned tasks effectively and efficiently. Consequently, the demand for adaptable, morphing aircraft has gradually increased. Since the first manned flight, aircraft designers have been intrigued by the concept of variable-geometry aircraft. Previously, the ability to change shape was used only to modify flight characteristics. However, recent advancements in materials, sensors, and actuators have sparked interest in the development of morphing aircraft [3]. In the development process, complex trade-off analyses and multidisciplinary optimization are frequently employed. Effective actuator drive systems also permit morphing for direct flight control. As new concepts for morphing aircraft emerge, the demand for efficient control methods is increasing [4]. However, control system design frameworks continue to fail to maximize all possible benefits of morphing.

Conventional fixed-wing aircraft are designed for optimal flight performance within the intended flight conditions and specified missions at the expense of performance in other flight regimes. In contrast, the ability to change configurations allows the morphing aircraft to fly nearly optimally in a broader range of flight conditions. In particular, morphing wings can be used to generate aerodynamic control forces by controlling the airflow directly around a vehicle. This additional degree of freedom can be used (i) to accomplish a movement that is normally impossible with conventional control surfaces alone, (ii) to increase agility and maneuverability when paired with conventional control surfaces, or (iii) to partially replace conventional control surfaces in the event of actuator failure. During flight, morphing configurations can be optimized in real time to maximize system-level benefits such as maneuverability, energy efficiency, survivability, etc. Numerous studies have explored the benefits of morphing, but few have focused on the control system that enables its full use.

It is difficult to use morphing parameters as control inputs in designing the control system of a morphing aircraft for two primary reasons. First, conventional control surfaces generate control torque for the roll, pitch, and yaw axes using long moment arms and minimal aerodynamic forces. However, wing morphing frequently generates forces and moments along multiple axes simultaneously. In addition, there is typically a significant nonlinear relationship between morphing parameters and the aerodynamic forces and torques that are generated. In a large-scale shape change, the location of the center of mass (CM), the moment of inertia (MOI), and the product of inertia (POI) can also vary considerably. Second, the majority of morphing actuators have a slower dynamic response to the command than conventional hydraulically actuated control surfaces. The low bandwidth of morphing actuators may significantly diminish the overall performance of control systems. Using the morphing parameters as control inputs necessitates careful consideration of the bandwidth gap.

If morphing parameters are considered exogenous parameters, the designed controller should be able to change its characteristics according to the changing morphing parameters. Since the dynamic characteristics change greatly depending on the morphing shape, it is generally difficult to ensure a sufficient level of stability and performance with a fixed controller. For example, control systems with traditional gain scheduling techniques cannot generally guarantee closed-loop stability when the scheduling parameter changes rapidly. In addition, interpolation or blending between point controllers involves a trial-and-error procedure with little theoretical guidance. Furthermore, in the region of transition between design points, robustness and performance guarantees of specific operating points are lost. Nevertheless, assuming that the parameters change slowly nullifies the fundamental advantages of morphing.

Historically, gain scheduling has been a common design technique for nonlinear flight control system design. A gain self-scheduled linear parameter-varying (LPV) control method has recently attracted interest as a suitable control method for morphing aircraft. Consequently, the majority of early studies employing LPV architecture involved flight control systems. The LPV framework enables the flight controller of a morphing aircraft to be designed with theoretical guarantees of robustness and performance across a wide variety of operating conditions and configurations. Incorporating the nonlinearity of the plant into scheduling parameters is also an effective method for addressing this nonlinearity. Because the LPV framework shares the basic control interconnection structure with a standard multivariable controller, such as a $H_{\infty }$ controller, different actuator bandwidths can be considered during the design process. In addition, the stability guarantee for arbitrarily fast parameter variations allows the morphing parameters to be used freely to the maximum without compromising stability.

The Wright Flyer, the first powered airplane, can be considered a morphing aircraft because it deforms its wings without a separate control surface [5]. Through the 1980s, various types of variable-geometry aircraft were developed primarily for military purposes. Then, beginning in the 1990s, modern morphing aircraft emerged, and morphing technology has recently been applied to small unmanned aerial vehicles. This trend is primarily attributable to the development of new materials, actuators, and sensors, as well as the fact that modern aircraft must perform significantly more complicated tasks than in the past [6].

Classical gain scheduling techniques have been widely adopted for systems with a wide range of operations. Control design methods based on linear time-invariant (LTI) models such as classical control, loop-shaping, $H_{2}$ (LQG) optimal control, $H_{\infty }$ suboptimal control, mixed-sensitivity control, and mu-synthesis can be applied. Various linear and classical control methods have been applied to control morphing aircraft, including proportional–integral–derivative (PID) control [7]. Many studies have adopted conventional gain scheduling techniques or switching methods [8,9]. Learning-based or data-driven approaches (reinforcement learning, neural-network-based adaptive control) [10,11,12] and adaptive approaches [12,13,14] have also been adopted to address the time-varying nature of morphing aircraft. Control allocation methods have been adopted to address an overactuated model of morphing aircraft from incorporating morphing parameters into a control input [15]. Various nonlinear control methods have also been applied, including sliding mode control [16,17], nonlinear dynamic inversion [18], backstepping control [19], and disturbance-observer-based control [20,21,22]. LPV and robust control methods are also extensively applied [23]. Many LPV techniques, such as switching LPV control, model predictive control based on the LPV model, data-driven strategies, and LPV control with scheduling uncertain parameters, have been applied to aerospace domain problems [24,25,26,27,28]. Fuzzy gain scheduling techniques have also been studied [29,30].

**Remark** **1.** *When comparing to other methods such as gain-scheduled $H_{\infty }$ control, nonlinear dynamic inversion control, and learning-based control, the LPV control method adopted in this study provides a rigorous stability guarantee under arbitrarily fast morphing, which requires moderate online computation for practical implementation. Note that, for an LPV controller to work properly, the LPV model must be able to successfully mimic the nonlinear model.*

In this study, LTI models of longitudinal and lateral-directional dynamics were obtained by linearizing the nonlinear model across all flight conditions and shape transformations. Interpolation was used to parameterize the family of pointwise LTI models to obtain the LPV models. The symmetric component of span variation was considered as a scheduling parameter alongside airspeed and altitude, while the asymmetric component was considered as a lateral-directional axis control input alongside ailerons and rudders. CAS and autopilots were designed for manual and automatic flight, respectively. The symmetric morphing parameters were used to assist the morphing aircraft in executing the commanded maneuver and improving the flight performance across a variety of flight conditions, while the asymmetric morphing parameter served as an additional control input for the lateral-directional axis. Numerical simulation of various maneuvers demonstrates that the maneuverability of a morphing aircraft can be improved by adjusting the symmetric morphing parameter appropriately. In addition, the proposed control system successfully follows the flight trajectory under rapid variation in flight conditions and extremely fast morphing, whereas the baseline controller with the same parameter settings rapidly loses closed-loop stability.

This study’s contribution can be divided into three parts. First, a high-fidelity model of an asymmetric variable-span morphing UAV was developed, from which, nonlinear and LPV morphing aircraft models were derived. The nominal model is presented first, followed by the damage model, from which, the morphing model was derived. Through Jacobian linearization, a pointwise LTI model was derived from the trim condition. By introducing scheduling parameters, an LPV model was constructed, which was then analyzed in the frequency domain and time domain to gain physical insight into the controller design. Second, the design of the control augmentation system is based on LPV methods that utilize variable span morphing parameters as the control input and scheduling parameters. Based on LPV techniques, a control augmentation system was designed for the morphing-assisted maneuvers of morphing UAVs. A normal acceleration CAS was used for controlling longitudinal motion, whereas a roll rate CAS was used for controlling lateral motion. The CAS employed symmetric and asymmetric morphing to enhance agility and maneuverability, respectively. The effectiveness of the proposed scheme is demonstrated through numerical simulations of push-over and pull-up maneuvers and a high-g turn. Finally, an autopilot for the morphing-assisted flight of morphing UAVs based on LPV methods was designed. Autopilots for airspeed and altitude were designed to control longitudinal motion. A roll angle autopilot was designed for controlling lateral motion. The autopilot employed symmetric and asymmetric morphing to enhance agility and maneuverability, respectively. The effectiveness of the proposed scheme is demonstrated using numerical simulations.

- A high-fidelity model of an asymmetric variable-span morphing UAV was developed, from which, nonlinear and LPV morphing aircraft models were derived.
- The design of the control augmentation system is based on LPV methods that utilize variable span morphing parameters as the control input and scheduling parameters.
- An autopilot for morphing-assisted flight of morphing UAVs based on LPV methods was designed.

**Remark** **2.** *This paper is focused on application rather than the theory itself. Many practical considerations are provided in the manuscript throughout the LPV modeling and control design procedure, which is vital in the control system design for morphing aircraft. In particular, the capability of symmetric and asymmetric morphing is fully exploited to maximize the performance of the morphing aircraft. Moreover, it is demonstrated in the simulation results that morphing can be used to either assist the maneuver or flight control.*

This paper is organized as follows. In Section 2, the LPV model of morphing aircraft is derived. In Section 3, LPV-based CAS is designed, and in Section 4, LPV-based autopilot is designed. In Section 5, a summary of the main results of this study and suggestions for future work are provided.

## 2. Asymmetric Variable-Span Morphing UAV Model

**Figure 1:**
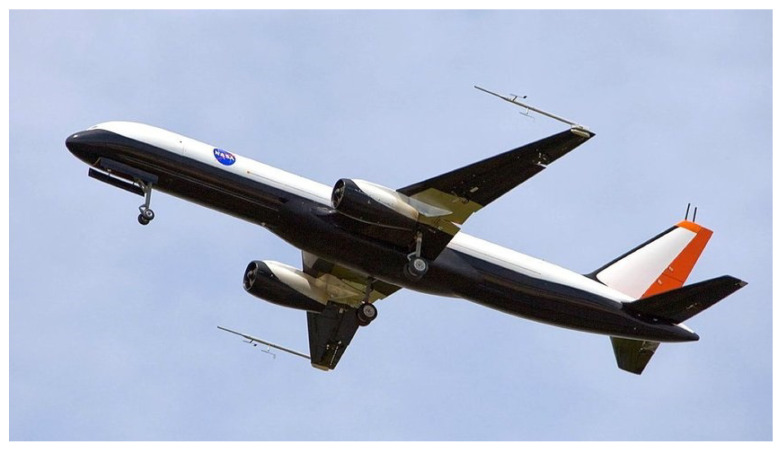
NASA GTM-T2 [31].

The NASA GTM shown in Figure 1 was selected as the baseline model for the development of a morphing UAV model in this study. The GTM simulation software is accessible via an open-source agreement with NASA [31]. GTM’s high-fidelity models of dynamics, aerodynamics, sensors, and actuators are ideal for not only analyzing the effects of morphing in flight but also demonstrating that the proposed technique can achieve the desired performance in a real system.

In the GTM, there are six damage cases, so a nonlinear variable-span morphing model can be derived for the scenario where 25% of the left wingtip is lost. It is possible to model the effects of variable-span morphing by assuming that the wingtips can telescope continuously rather than being lost. Therefore, the mass, CM, MOI, POI, and aerodynamic coefficients depend on the morphing parameters. It is possible to generate LTI models that decouple longitudinal and lateral-direction motions by linearizing the acquired nonlinear parameter variation model of the morphing UAV at each operational point. By examining the LTI model in the time and frequency domains, it is possible to acquire the necessary knowledge for controller design. By interpolating the LTI model family, the LPV model can be constructed. It can be demonstrated that the LPV model adequately captures the nonlinearity of the original system.

### 2.1. Nonlinear Model of a Morphing UAV

A nonlinear model of a morphing UAV was derived. First, the motion equations of the nominal model, aerodynamic model, sensor model, and actuator model are described in detail. In the case of asymmetric variable-span morphing, the CM, inertia matrix, and aerodynamic coefficients were then obtained as functions of the morphing parameters.

#### 2.1.1. Nominal Model of a Baseline Model

Before presenting the morphing aircraft model, the construction process for the baseline model is described. The motion equations of a morphing UAV are identical to those of a conventional fixed-wing aircraft. The following states were utilized directly in numerical simulations.

Flat-Earth and constant gravitational acceleration were assumed in navigation equations. The geographic coordinate system was used to generate the vehicle’s position in the north-east-down (NED) coordinate system based on the world geodetic system 1984 (WGS84), which was used only for visualizing the vehicle’s spatial trajectory. The geodetic coordinate navigation equations can be represented as

(1) $$\begin{array}{cc} \dot{\varphi } & =\frac{V_{N}}{R_{0}+h} \end{array}$$

(2) $$\begin{array}{cc} \dot{\lambda } & =\frac{V_{E}\sec L}{R_{0}+h} \end{array}$$

(3) $$\begin{array}{cc} \dot{h} & =-V_{D} \end{array}$$

(4) $$\left[\begin{array}{c} V_{N}\\ V_{E}\\ V_{D} \end{array}\right]=\left[\begin{array}{ccccc} c\theta c\psi & \; & -c\phi s\psi +s\phi s\theta c\psi & \; & s\phi s\psi +c\phi s\theta c\psi \\ c\theta s\psi & \; & c\phi c\psi +s\phi s\theta s\psi & \; & -s\phi c\psi +c\phi s\theta s\psi \\ -s\theta & \; & s\phi c\theta & \; & c\phi c\theta \end{array}\right]\left[\begin{array}{c} U\\ V\\ W \end{array}\right]$$

where φ is the geodetic latitude, λ is the longitude, *h* is the height above ground, $R_{0}$ is the Earth’s radius, $V_{N}$, $V_{E}$, and $V_{D}$ are the north, east, and down components of the velocity vector in the local geographic coordinate system, respectively, *U*, *V*, and *W* are the x-axis, y-axis, and z-axis components of the velocity vector in the body-fixed coordinate system, respectively, ϕ, θ, and ψ are the Euler angles (roll, pitch, and yaw angles) of the vehicle body axes relative to the geographic system, respectively, and *s* and *c* stand for the sine and cosine, respectively. The forward-right-down system was utilized for the body-fixed coordinate system, whose origin is fixed at the vehicle’s CM.

Assuming constant gravitational acceleration, the force equations can be represented as [32]

(5) $$\begin{array}{cc} \dot{U} & =RV-QW+\frac{X_{G}+X_{A}+X_{T}}{m} \end{array}$$

(6) $$\begin{array}{cc} \dot{V} & =-RU+PW+\frac{Y_{G}+Y_{A}+Y_{T}}{m} \end{array}$$

(7) $$\begin{array}{cc} \dot{W} & =QU-PV+\frac{Z_{G}+Z_{A}+Z_{T}}{m} \end{array}$$

where

(8) $$\begin{array}{cc} X_{G} & =-g\sin\theta \end{array}$$

(9) $$\begin{array}{cc} Y_{G} & =g\sin\phi \cos\theta \end{array}$$

(10) $$\begin{array}{cc} Z_{G} & =g\cos\phi \cos\theta \end{array}$$

and *P*, *Q*, and *R* are the x-axis, y-axis, and z-axis components of the angular velocity vector in the body-fixed coordinate system, respectively, $X_{G}$, $X_{A}$, $X_{T}$, $Y_{G}$, $Y_{A}$, $Y_{T}$, $Z_{G}$, $Z_{A}$, and $Z_{T}$ are the x-axis, y-axis, and z-axis components of the gravity, aerodynamic force, and engine thrust vectors in the body-fixed coordinate system, respectively, *g* is the gravitational acceleration, and m is the vehicle mass.

The kinematic equations of the Euler angle dynamics can be represented as

(11) $$\begin{array}{cc} \dot{\phi } & =P+\tan\theta (Q\sin\phi +R\cos\phi ) \end{array}$$

(12) $$\begin{array}{cc} \dot{\theta } & =Q\cos\phi -R\sin\phi \end{array}$$

(13) $$\begin{array}{cc} \dot{\psi } & =\frac{\left(Q\sin\phi +R\cos\phi \right)}{\cos\theta } \end{array}$$

The moment equations can be represented as

(14) $$\left[\begin{array}{c} P\\ Q\\ R \end{array}\right]=J^{-1}\left(\left[\begin{array}{c} l_{A}+l_{T}\\ m_{A}+m_{T}\\ n_{A}+n_{T} \end{array}\right]-\left[\begin{array}{c} P\\ Q\\ R \end{array}\right]\times \left(J\left[\begin{array}{c} P\\ Q\\ R \end{array}\right]\right)\right)$$

where $l_{A}$, $l_{T}$, $m_{A}$, $m_{T}$, $n_{A}$, and $n_{T}$ are the x-axis, y-axis, and z-axis components of the aerodynamic torque vector and the torque vector generated by the engine about the CM in the body-fixed coordinate system, respectively, and *J* is the inertia matrix of the vehicle with respect to the body-fixed coordinate system. Because the motion equations are formulated with respect to the CM, gravitational moments are always zero.

NASA GTM aerodynamic data were utilized directly, with the aerodynamic coefficients provided as data lookup tables of aerodynamic angles. Dependency on altitude is accounted for by the atmospheric density term, which is derived from the 1976 US standard atmosphere. The dynamic pressure term includes airspeed dependence. The GTM is a medium-sized (57.71 lb) unmanned aerial vehicle that flies in the subsonic region (Mach 0.09–0.18), with compressibility effects perhaps being negligible below Mach 0.3. Therefore, it may be claimed that the GTM is capable of simulating with appropriate accuracy.

The aerodynamic force components can be represented as

(15) $$\begin{array}{cc} X_{A} & =\bar{q}SC_{X} \end{array}$$

(16) $$\begin{array}{cc} Y_{A} & =\bar{q}SC_{Y} \end{array}$$

(17) $$\begin{array}{cc} Z_{A} & =\bar{q}SC_{Z} \end{array}$$

where $\bar{q}=0.5\rho V_{T}^{2}$ is the dynamic pressure, *S* is the planform area of the main wing, ρ is the atmospheric density, $V_{T}$ is the true airspeed (TAS), and $C_{X}$, $C_{Y}$, and $C_{Z}$ are body-axis aerodynamic force coefficients.

The aerodynamic torque components can be represented as

(18) $$\left[\begin{array}{c} l_{A}\\ m_{A}\\ n_{A} \end{array}\right]=\left[\begin{array}{c} \bar{q}SbC_{l}\\ \bar{q}S\bar{c}C_{m}\\ \bar{q}SbC_{n} \end{array}\right]+\left(r_{cp}-r_{cm}\right)\times \left[\begin{array}{c} X_{A}\\ Y_{A}\\ Z_{A} \end{array}\right]$$

where *b* is the wing span, $\bar{c}$ is the mean aerodynamic chord (MAC), $C_{l}$, $C_{m}$, and $C_{n}$ are body-axis aerodynamic moment coefficients, respectively, and $r_{cp}$ and $r_{cm}$ are the position vectors of the center of pressure (25% MAC) and the CM (21.99% MAC) in the ARS.

The coefficient vector $C={\left[C_{X}\;C_{Y}\;C_{Z}\;C_{l}\;C_{m}\;C_{n}\right]}^{T}$ consists of the basic airframe components $C_{\mathrm{ba}}$, control surface components $C_{\mathrm{cs}}$, dynamic derivative components $C_{\mathrm{dd}}$, and stall-rolling moment asymmetry components $C_{\mathrm{rma}}$. The basic airframe component is given in the form of a data lookup table that depends on the angle of attack (AOA) and the angle of sideslip (AOS). The control surface components also depend on the corresponding control surface deflections. The aerodynamic coefficients are built up as [31]

(19) $$C=C_{\mathrm{ba}}+\Delta C_{\mathrm{cs}}+\Delta C_{\mathrm{dd}}+\Delta C_{\mathrm{rma}}$$

Five sensor systems are included in the GTM.

- Air data system (ADS)
- MIDG II GPS/INS (MIDG)
- MAG3 6-DOF analog IMU with a triaxial magnetometer, accelerometer, and gyro (MAG3)
- Surface potentiometers (Pots)
- Engine control unit (ECU)

The ADS provides the barometric altitude, AOA, AOS, dynamic pressure, TAS, and temperature ratio. At a sampling rate of 50 Hz, the MIDG provides Euler angles, geodetic latitude, longitude, altitude, and geographical velocity. MAG3 offers 3-axis acceleration and angular velocity. The pots provide control surface deflections, and the ECU provides data on the left and right engines’ internal operations. Sensor measurements are corrupted by appropriate dynamics, scale factor error, bias, white noise, saturation, truncation error, and transport delay.

The control surface models for elevators, ailerons, rudders, spoilers, and flaps include the rate limit, saturation, transport delay, and an optional dead zone with the following first-order dynamics and 5 Hz bandwidth (BW).

(20) $$G_{cs}\left(s\right)=\frac{10\pi }{s+10\pi }$$

Two Jetcat P70 engines were modeled, including throttle-to-RPM nonlinearity (as a cubic polynomial), RPM-to-thrust nonlinearity (as a cubic polynomial), a ram drag model, the effect of atmospheric pressure, and saturation with the first-order lag model shown below.

(21) $$G_{eng}\left(s\right)=\frac{-0.1474s+0.7314}{s^{2}+1.336s+0.7314}$$

The fuel flow rate is a parabolic function of the engine speed.The gyroscopic effect of the rotating rotor, off-axis thrust, torque generated by a thrust that is not aligned with the CM, and differential thrust is also accounted for.

#### 2.1.2. Morphing UAV Model

Based on the nominal UAV model, a morphing UAV model was derived. The GTM includes six damage cases:

- Rudder Off;
- Vertical Tail Off;
- Left Outboard Flap Off;
- Left Wingtip Off;
- Left Elevator Off;
- Left Stabilizer Off.

Except for the fourth case, it corresponds to the loss of a control surface. Case 4 involves the loss of the outboard (approximately 25% of the semispan) left wingtip. The primary aerodynamic effect is a rolling moment bias that causes the left wing to drop (-DeltadCl), and this effect grows as alpha increases. Due to the location of the ailerons on the wingtip, the original model cannot use ailerons. In this study, however, it was assumed that the wingtip can telescope and the aileron can still function as it was installed slightly further inboard.

The effect of the roll axis moment can be maintained if the size of the aileron is increased proportionally to its proximity to the centerline. In this manner, the magnitude of the force generated by the ailerons increases; however, when the ailerons are used in opposite directions, the majority of the force is canceled out. It was assumed that the right wing could also telescope, similar to the left wing. Using the changes in mass, inertia matrix, and aerodynamic coefficient when the tip of the left wing is removed, it is possible to model the mass, inertia matrix, and aerodynamic coefficient when the left and right wings are adjusted independently. In this study, the left and right morphing parameters etal and etar were defined, representing the length variation ratio of each wing between −25% and 0%. The variables for symmetric and asymmetric morphing were then defined as follows:

(22) $$\begin{array}{cc} \eta_{s} & =\frac{\eta_{l}+\eta_{r}}{2} \end{array}$$

(23) $$\begin{array}{cc} \eta_{a} & =\frac{\eta_{l}-\eta_{r}}{2} \end{array}$$

It was also assumed that the differences in aerodynamic coefficients between the shortest and longest variable spans can be approximated by linear interpolation with acceptable error. Note that the CM and inertia matrices can be obtained precisely using the model data. It can be demonstrated that the shift in the center of gravity is a linear function of the morphing variables, whereas the MOIs and POIs are parabolic functions.

Variations in the mass $\Delta_{d}m$, CM $\Delta_{d}r_{cm}$, inertia matrix $\Delta_{d}J$, and aerodynamic coefficients $\Delta_{d}C$ are known in the damaged configuration. Variations in the mass Δm, CM $\Delta r_{cm}$, inertia matrix ΔJ, and aerodynamic coefficients ΔC can be represented as functions of the morphing parameters in the morphed configuration. The quasistatic assumption was adopted in this study, and the unsteady effect during the morphing process was not modeled because the original model does not include this effect.

The aircraft can be divided into three components: the moving left wingtip (subscript *l*), stationary main body (subscript *b*), and moving right wingtip (subscript *r*). The CM variations in the left and right wingtips in the morphed configuration can be represented as

(24) $$\Delta r_{l}=-\frac{\eta_{l}}{25}\left[\begin{array}{c} \frac{b\tan\Lambda }{8}\;\\ \frac{b}{8}\;\\ \frac{b\tan\Gamma }{8\cos\Lambda } \end{array}\right],\;\Delta r_{r}=-\frac{\eta_{r}}{25}\left[\begin{array}{c} \frac{b\tan\Lambda }{8}\;\\ -\frac{b}{8}\;\\ \frac{b\tan\Gamma }{8\cos\Lambda } \end{array}\right]$$

where Λ is the leading edge sweep angle and Γ is the wing dihedral angle. The gross CM variation in the morphed configuration can be represented as

(25) $$\Delta r_{cm}=\left(\frac{m_{l}}{m}\right)\Delta r_{l}+\left(\frac{m_{r}}{m}\right)\Delta r_{r}$$

where $m_{l}$ and $m_{r}$ are the masses of the left and right wingtips, respectively. Finally, the morphed gross CM can be represented as

(26) $$r_{cm}={\bar{r}}_{cm}+\Delta r_{cm}$$

Note that

(27) $$m_{l}=m_{r}=\Delta_{d}m$$

and

(28) $$m=m_{l}+m_{b}+m_{r}$$

where $m_{b}$ is the main body mass.

The nominal gross CM can be represented as

(29) $$m{\bar{r}}_{cm}=m_{l}{\bar{r}}_{l}+m_{b}{\bar{r}}_{b}+m_{r}{\bar{r}}_{r}$$

where ${\bar{r}}_{l}$, ${\bar{r}}_{b}$, and ${\bar{r}}_{l}$ are the nominal CM of the left wingtip, main body, and right wingtip, respectively. Note that

(30) $${\bar{r}}_{r}=\left[\begin{array}{ccccc} 1 & \; & 0 & \; & 0\\ 0 & \; & -1 & \; & 0\\ 0 & \; & 0 & \; & 1 \end{array}\right]{\bar{r}}_{l}$$

The damaged gross CM can be represented as

(31) $$\left(m-m_{l}\right)\left({\bar{r}}_{cm}+\Delta_{d}r_{cm}\right)=m_{b}{\bar{r}}_{b}+m_{r}{\bar{r}}_{r}$$

Subtracting Equation (31) from Equation (29) yields ${\bar{r}}_{l}$ as

(32) $$m_{l}{\bar{r}}_{cm}-\left(m-m_{l}\right)\Delta_{d}r_{cm}=m_{l}{\bar{r}}_{l}$$

which can be rewritten as

(33) $${\bar{r}}_{l}={\bar{r}}_{cm}-\left(\frac{m}{m_{l}}-1\right)\Delta_{d}r_{cm}$$

Now, ${\bar{r}}_{r}$ can be obtained by Equation (30). Then, ${\bar{r}}_{b}$ can be calculated by either Equation (29) or Equation (31). The components’ morphed CM can be represented as

(34) $$r_{i}={\bar{r}}_{i}+\Delta r_{i},\;i=l,b,r$$

By applying the parallel axis theorem, also known as the Huygens–Steiner theorem, the nominal and morphed gross inertia matrix can be represented as

(35) $$\bar{J}={\bar{J}}_{l}-m_{l}{\left[{\bar{r}}_{l}-{\bar{r}}_{cm}\right]}^{2}+{\bar{J}}_{b}-m_{b}{\left[{\bar{r}}_{b}-{\bar{r}}_{cm}\right]}^{2}+{\bar{J}}_{r}-m_{r}{\left[{\bar{r}}_{r}-{\bar{r}}_{cm}\right]}^{2}$$

(36) $$J={\bar{J}}_{l}-m_{l}{\left[r_{l}-r_{cm}\right]}^{2}+{\bar{J}}_{b}-m_{b}{\left[r_{b}-r_{cm}\right]}^{2}+{\bar{J}}_{r}-m_{r}{\left[r_{r}-r_{cm}\right]}^{2}$$

where ${\bar{J}}_{l}$, ${\bar{J}}_{b}$, and ${\bar{J}}_{r}$ are the inertia matrices about their own centers of mass. Note that [r] is a skew-symmetric matrix associated with $r={\left[x\;y\;z\right]}^{T}$ as

(37) $$\left[r\right]=\left[\begin{array}{ccccc} 0 & \; & -z & \; & y\\ z & \; & 0 & \; & -x\\ -y & \; & x & \; & 0 \end{array}\right]$$

The following equation can be useful:

(38) $$-{\left[r\right]}^{2}=\left[\begin{array}{ccccc} y^{2}+z^{2} & \; & -xy & \; & -xz\\ -yx & \; & x^{2}+z^{2} & \; & -yz\\ -zx & \; & -xy & \; & x^{2}+y^{2} \end{array}\right]=\mathrm{tr}\left(rr^{T}\right)I_{3}-rr^{T}$$

Finally, the inertia matrix variation due to morphing can be obtained by subtracting Equation (35) from Equation (36) as

(39) $$\begin{array}{cc} \Delta J= & m_{l}{\left[{\bar{r}}_{l}-{\bar{r}}_{cm}\right]}^{2}+m_{b}{\left[{\bar{r}}_{b}-{\bar{r}}_{cm}\right]}^{2}+m_{r}{\left[{\bar{r}}_{r}-{\bar{r}}_{cm}\right]}^{2}\\ - & m_{l}{\left[r_{l}-r_{cm}\right]}^{2}-m_{b}{\left[r_{b}-r_{cm}\right]}^{2}-m_{r}{\left[r_{r}-r_{cm}\right]}^{2} \end{array}$$

**Figure 2:**
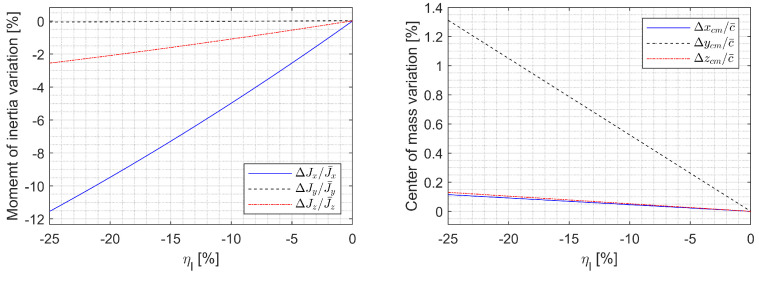
Parameter variation due to $\eta_{l}$ when $\eta_{r}=0$%.

**Figure 3:**
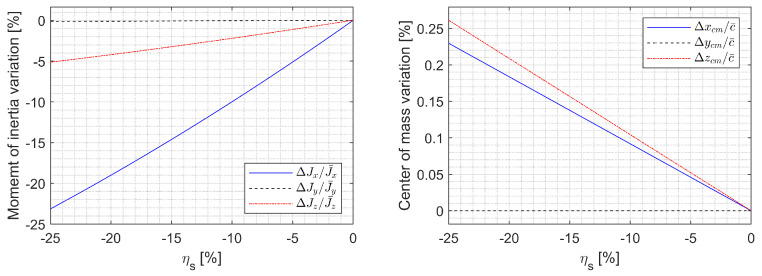
Parameter variation due to $\eta_{s}$ when $\eta_{a}=0$%.

The parameter variations due to $\eta_{l}$ and $\eta_{s}$ are shown in Figure 2 and Figure 3. The x-axis moment of inertia can be varied up to 23%, which means that the roll rate can be increased up to 30% by retracting the span due to angular momentum conservation. The CM can be shifted up to 1.3% MAC off the plane of symmetry by asymmetric morphing. Note that the CM moving back and forth up to 0.23% MAC due to a nonzero sweep may affect longitudinal stability, where the nominal static margin is 3.01% MAC.

In the variable-span morphing wing, the lift coefficient is nearly linearly proportional to the span [33] because the area of the lifting surface is linearly proportional to the span. The total drag in the subsonic flow consists of the profile drag and the induced drag. The drag coefficient for the induced drag of a high-aspect lightly swept wing in subsonic flow can be modeled as follows [34]:

(40) $$C_{D_{i}}=\frac{C_{L}^{2}}{\pi eAR}$$

where *e* is the efficiency factor, which is close to unity, and AR is the aspect ratio defined as follows:

$$AR=\frac{b^{2}}{S}$$

Therefore, the induced drag also varies linearly with respect to $\eta_{s}$. When an aircraft’s wing is not stalled, the aircraft’s parasite drag is nearly entirely composed of skin friction [32]. The quantity of skin friction drag depends on the aircraft’s wetted area. Therefore, the parasite drag and, in turn, the total drag can also be assumed to linearly vary with respect to $\eta_{s}$.

Similarly, the coefficients for the pitching moment and crosswind force can be assumed to vary linearly. In contrast to the longitudinal and force coefficients, the rolling and yawing moment coefficients vary in a parabola with a span due to the linear variation in the moment arm. However, if the moving portion of the span is relatively short compared to the entire wing, the effect of variation at the moment arm may be negligible. Therefore, the morphing-induced increase in the rolling and yawing moment coefficients can also be assumed to be proportional to the morphing parameter.

The aerodynamic coefficient variation due to left span morphing can be represented as

(41) $$\Delta_{l}C\left(\alpha ,\beta \right)=-\left(\frac{\eta_{l}}{25}\right)\Delta_{d}C\left(\alpha ,\beta \right)$$

where $\Delta_{d}C$ is the aerodynamic coefficient variation due to left wingtip loss. Note that

(42) $$\Delta_{r}C\left(\alpha ,\beta \right)=\mathrm{diag}\left(1,-1,1,-1,1,-1\right)\Delta_{l}C\left(\alpha ,-\beta \right)$$

Finally, the aerodynamic coefficient variation in the morphed configuration can be represented as

(43) $$\Delta C\left(\alpha ,\beta \right)=\Delta_{l}C\left(\alpha ,\beta \right)+\Delta_{r}C\left(\alpha ,\beta \right)$$

When β≈0 in Equation (42),

(44) $$\begin{array}{cc} \Delta_{r}C_{lon} & =\Delta_{l}C_{lon} \end{array}$$

(45) $$\begin{array}{cc} \Delta_{r}C_{lat} & =-\Delta_{l}C_{lat} \end{array}$$

where $C_{lon}={\left[C_{X}\;C_{Z}\;C_{m}\right]}^{T}$ and $C_{lat}={\left[C_{Y}\;C_{l}\;C_{n}\right]}^{T}$. Then,

(46) $$\begin{array}{cc} \Delta C_{lon} & =-2\left(\frac{\eta_{s}}{25}\right)\Delta_{d}C \end{array}$$

(47) $$\begin{array}{cc} \Delta C_{lat} & =-2\left(\frac{\eta_{a}}{25}\right)\Delta_{d}C \end{array}$$

**Figure 4:**
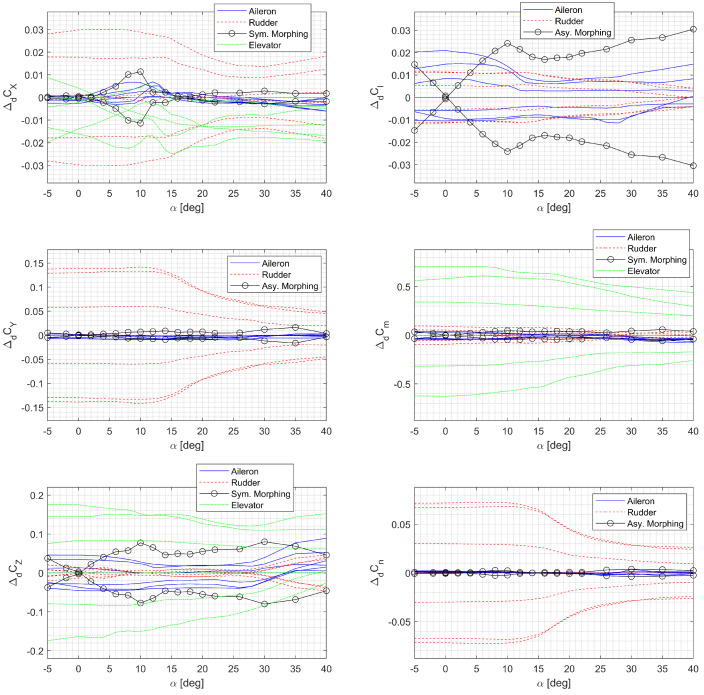
Control surface increment to the aerodynamic coefficients when β=0 deg.

Note that the longitudinal motion is affected by symmetric morphing, whereas the lateral-directional motion is affected by asymmetric morphing. If the configuration where $\eta_{l}=\eta_{r}=-12.5$% is selected as a nominal configuration, the maximum variation in the aerodynamic coefficients becomes $\pm \Delta_{d}C$. The morphing parameters are suitable for the control input because they appear affinely in the forces and moments. The increments are nearly linearly proportional in the prestall region where α∈[−510] deg, while the conventional control surface increments are only lightly affected by the AOA, as shown in Figure 4. Consequently, the control effectiveness of asymmetric morphing in the roll axis is low when the AOA is small. However, the effectiveness of asymmetric morphing becomes even greater than the ailerons in the poststall region (α>10 deg), as shown in Figure 4. Furthermore, symmetric morphing can be used to delay stall in low-speed flights or high-g maneuvers by increasing the lift coefficient, which, in turn, decreases the AOA toward the prestall region while generating the same lift.

**Figure 5:**
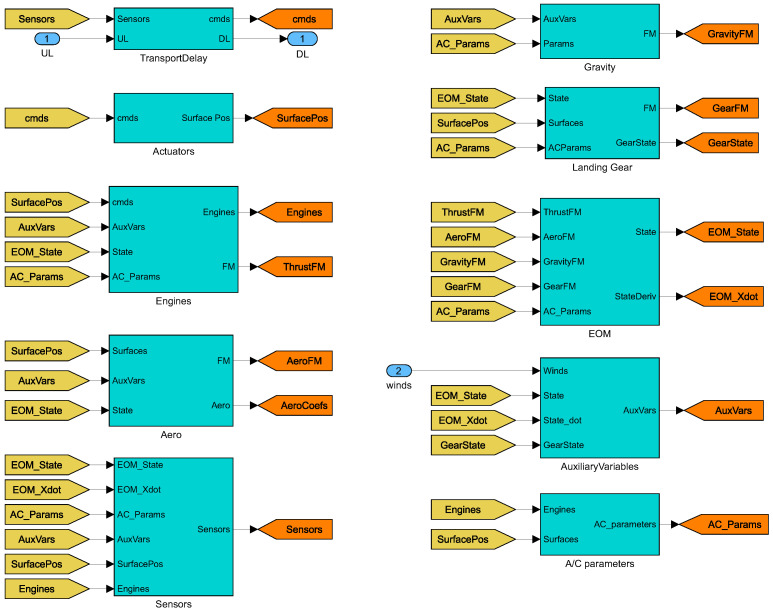
Building blocks of GTM.

The nonlinear model used in the following simulation scenarios is shown in Figure 5.

### 2.2. Derivation of an LPV Model of a Morphing UAV

#### 2.2.1. Trim Analysis and Scheduling Parameter Selection

In this study, the scheduling parameters were airspeed and altitude. In addition, the parameter for symmetric morphing was included in the scheduling parameters because it has significant effects on longitudinal aerodynamics, which affects the overall flight performance. The asymmetric morphing parameter has a minimal effect on the longitudinal motion, but it can be utilized as a control input for the lateral motion.

**Figure 6:**
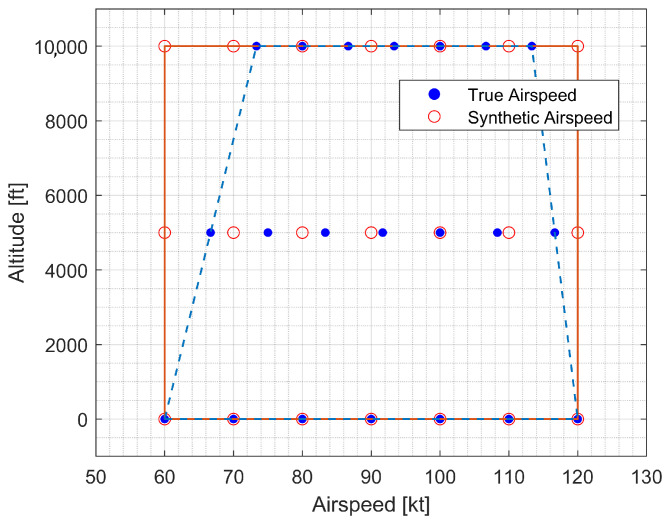
Rectangular scheduling parameter domain.

Note that a rectangular parameter grid is necessary for the LPV controller design. However, the trimmable region of the GTM appears triangular in the TAS versus altitude diagram. The service ceiling is approximately 30,000 feet, where the trimmable TAS converges to 100 knots. Therefore, a new speed-related parameter must be introduced so that the parameter domain becomes rectangular. In this study, the following synthetic airspeed was introduced [35]:

(48) $$V_{S}=V_{0}-\frac{h_{0}}{h_{0}-h}\left(V_{0}-V_{T}\right),\;h<h_{0}$$

where $V_{0}$ = 100 kt and $h_{0}$ = 30,000 ft. Note that Equation (48) becomes singular when $h=h_{0}$. However, the normal operating altitude of medium-size UAVs such as GTM is much lower than 30,000 ft. Then, the flight envelope becomes a rectangle, as shown in Figure 6.

Now, the scheduling parameter vector $\rho ≜{\left[V\;h\;\eta_{s}\right]}^{T}$ with three parameters is considered. The considered parameter grid is

- $V_{S}\in \left[60,70,80,90,100,110,120\right]$ in kt;
- h∈[0,5000,10,000] in ft;
- $\eta_{s}\in \left[-25,-12.5,0\right]$ in %.

As a result of the trim analysis, the true airspeed that can satisfy the trim condition at sea level is between approximately 60 kt and 120 kt. This trim airspeed range tends to narrow on both sides as the altitude increases, which appears constant even when the altitude changes if the synthetic airspeed is introduced. A 10 kt airspeed interval was set to properly reflect the nonlinear dependence of the aerodynamic force and torque. Atmospheric density changes as altitude changes, leading to nonlinear changes in dynamic pressure. The altitude was set at 5000 ft intervals because the nonlinearity is not strong in the atmospheric density curve. In the case of the morphing shape, the models at both ends were given from the nominal and damaged models, and the intermediate shape point was added to the parameter domain.

**Remark** **3.** *The range of speed was set within the range where the GTM could maintain its trim state. The range of altitude is the normal operational altitudes. The range of the symmetric morphing parameter is the range provided by the damage model. If the number of the grid points is increased, the computation time may become excessive or a feasible solution may not be found. The interval of each variable was set to an appropriate value that captures enough nonlinearity but does not overly increase the total number of the grid points. Whether it is dense enough can be verified by comparing the simulation results for the LPV model and the nonlinear model or by investigating the results of the nonlinear simulation.*

#### 2.2.2. Lpv Modeling

The motion equations can be decoupled into equations describing the longitudinal motion and lateral-directional motion. The LTI models for the decoupled motions can be obtained at each parameter grid through Jacobian linearization. The wind axis was chosen in this study because of its advantages in decoupling.

The wind-axis longitudinal motion equations can be expressed as

(49) $$\begin{array}{cc} \left[\begin{array}{c} {\dot{V}}_{T}\\ \dot{\alpha }\\ \dot{q}\\ \dot{\theta } \end{array}\right] & =\left[\begin{array}{ccccccc} X_{V}+X_{T_{V}}\cos\left(\alpha_{0}+\alpha_{T}\right) & \; & X_{\alpha } & \; & 0 & \; & -g\cos\gamma_{0}\\ \frac{Z_{V}-Z_{T_{V}}\sin\left(\alpha_{0}+\alpha_{T}\right)}{V_{0}} & \; & \frac{Z_{\alpha }}{V_{0}} & \; & 1+\frac{Z_{q}}{V_{0}} & \; & -\frac{g\sin\gamma_{0}}{V_{0}}\\ M_{V}+M_{T_{V}} & \; & M_{\alpha }+M_{T_{\alpha }} & \; & M_{q} & \; & 0\\ 0 & \; & 0 & \; & 1 & \; & 0 \end{array}\right]\left[\begin{array}{c} V_{T}\\ \alpha \\ q\\ \theta \end{array}\right]\\ & +\left[\begin{array}{ccc} X_{\delta_{t}}\cos\left(\alpha_{0}+\alpha_{T}\right) & \; & X_{\delta_{e}}\\ -\frac{X_{\delta_{t}}\sin\left(\alpha_{0}+\alpha_{T}\right)}{V_{0}} & \; & \frac{Z_{\delta_{e}}}{V_{0}}\\ M_{\delta_{t}} & \; & M_{\delta_{e}}\\ 0 & \; & 0 \end{array}\right]\left[\begin{array}{c} \delta_{t}\\ \delta_{e} \end{array}\right] \end{array}$$

The short-period mode dynamics can be extracted by eliminating the phugoid mode dynamics as

(50) $$\left[\begin{array}{c} \dot{\alpha }\\ \dot{q} \end{array}\right]=\left[\begin{array}{ccc} \frac{Z_{\alpha }}{V_{0}} & \; & 1+\frac{Z_{q}}{V_{0}}\\ M_{\alpha }+M_{T_{\alpha }} & \; & M_{q} \end{array}\right]\left[\begin{array}{c} \alpha \\ q \end{array}\right]+\left[\begin{array}{c} X_{\delta_{e}}\\ \frac{Z_{\delta_{e}}}{V_{0}} \end{array}\right]\delta_{e}$$

The short-period mode dynamic was used for a longitudinal CAS design. The z-axis acceleration can be augmented to the measurement equation as

(51) $$\left[\begin{array}{c} \alpha \\ q\\ a_{z} \end{array}\right]=\left[\begin{array}{ccc} 1 & \; & 0\\ 0 & \; & 1\\ Z_{\alpha } & \; & 0 \end{array}\right]\left[\begin{array}{c} \alpha \\ q \end{array}\right]+\left[\begin{array}{c} 0\\ 0\\ Z_{\delta_{e}} \end{array}\right]\delta_{e}$$

Note that the transfer function from $\delta_{e}$ to $a_{z}$ has a nonminimum phase (NMP) zero. In this case, the initial response to a step input may have the opposite sign to the final response. The altitude dynamics can be augmented to the longitudinal dynamics as

(52) $$\begin{array}{cc} \left[\begin{array}{c} {\dot{V}}_{T}\\ \dot{\alpha }\\ \dot{q}\\ \dot{\theta }\\ \dot{h} \end{array}\right] & =\left[\begin{array}{ccccccccc} X_{V}+X_{T_{V}}\cos\left(\alpha_{0}+\alpha_{T}\right) & \; & X_{\alpha } & \; & 0 & \; & -g\cos\gamma_{0} & \; & 0\\ \frac{Z_{V}-Z_{T_{V}}\sin\left(\alpha_{0}+\alpha_{T}\right)}{V_{0}} & \; & \frac{Z_{\alpha }}{V_{0}} & \; & 1+\frac{Z_{q}}{V_{0}} & \; & -\frac{g\sin\gamma_{0}}{V_{0}} & \; & 0\\ M_{V}+M_{T_{V}} & \; & M_{\alpha }+M_{T_{\alpha }} & \; & M_{q} & \; & 0 & \; & 0\\ 0 & \; & 0 & \; & 1 & \; & 0 & \; & 0\\ 0 & \; & -V_{0} & \; & 0 & \; & V_{0} & \; & 0 \end{array}\right]\left[\begin{array}{c} V_{T}\\ \alpha \\ q\\ \theta \\ h \end{array}\right]\\ & +\left[\begin{array}{ccc} X_{\delta_{t}}\cos\left(\alpha_{0}+\alpha_{T}\right) & \; & X_{\delta_{e}}\\ -\frac{X_{\delta_{t}}\sin\left(\alpha_{0}+\alpha_{T}\right)}{V_{0}} & \; & \frac{Z_{\delta_{e}}}{V_{0}}\\ M_{\delta_{t}} & \; & M_{\delta_{e}}\\ 0 & \; & 0\\ 0 & \; & 0 \end{array}\right]\left[\begin{array}{c} \delta_{t}\\ \delta_{e} \end{array}\right] \end{array}$$

The augmented dynamics were used for the longitudinal autopilot design.

For the control of the lateral-directional motion, the asymmetric morphing parameter was included in the control input. The lateral-directional dynamics, where the differential thrust is not considered, can be represented as

(53) $$\left[\begin{array}{c} \dot{\beta }\\ \dot{p}\\ \dot{r}\\ \dot{\phi } \end{array}\right]=\left[\begin{array}{ccccccc} \frac{Y_{\beta }}{V_{0}} & \; & \frac{Y_{p}}{V_{0}} & \; & \frac{Y_{r}}{V_{0}}-1 & \; & \frac{g\cos\theta_{0}}{V_{0}}\\ L_{\beta } & \; & L_{p} & \; & L_{r} & \; & 0\\ N_{\beta } & \; & N_{p} & \; & N_{r} & \; & 0\\ 0 & \; & \frac{\cos\gamma_{0}}{\cos\theta_{0}} & \; & \frac{\sin\gamma_{0}}{\cos\theta_{0}} & \; & 0 \end{array}\right]\left[\begin{array}{c} \beta \\ p\\ r\\ \phi \end{array}\right]+\left[\begin{array}{ccccc} \frac{Y_{\delta_{a}}}{V_{0}} & \; & \frac{Y_{\delta_{r}}}{V_{0}} & \; & \frac{Y_{\eta_{a}}}{V_{0}}\\ L_{\delta_{a}} & \; & L_{\delta_{r}} & \; & L_{\eta_{a}}\\ N_{\delta_{a}} & \; & N_{\delta_{r}} & \; & N_{\eta_{a}}\\ 0 & \; & 0 & \; & 0 \end{array}\right]\left[\begin{array}{c} \delta_{a}\\ \delta_{r}\\ \eta_{a} \end{array}\right]$$

Equation (53) was used for the lateral-directional autopilot design. When the airspeed is sufficiently high, the roll angle dynamics become negligible. In this case, the roll angle dynamics can be eliminated as

(54) $$\left[\begin{array}{c} \dot{\beta }\\ \dot{p}\\ \dot{r} \end{array}\right]=\left[\begin{array}{ccccc} \frac{Y_{\beta }}{V_{0}} & \; & \frac{Y_{p}}{V_{0}} & \; & \frac{Y_{r}}{V_{0}}-1\\ L_{\beta } & \; & L_{p} & \; & L_{r}\\ N_{\beta } & \; & N_{p} & \; & N_{r} \end{array}\right]\left[\begin{array}{c} \beta \\ p\\ r \end{array}\right]+\left[\begin{array}{ccccc} \frac{Y_{\delta_{a}}}{V_{0}} & \; & \frac{Y_{\delta_{r}}}{V_{0}} & \; & \frac{Y_{\eta_{a}}}{V_{0}}\\ L_{\delta_{a}} & \; & L_{\delta_{r}} & \; & L_{\eta_{a}}\\ N_{\delta_{a}} & \; & N_{\delta_{r}} & \; & N_{\eta_{a}} \end{array}\right]\left[\begin{array}{c} \delta_{a}\\ \delta_{r}\\ \eta_{a} \end{array}\right]$$

Equation (54) was used for the lateral-directional CAS design. By applying interpolation to the family of LTI models, an LPV model can be obtained.

## 3. Cas Design Based on the LPV Method for Morphing-Assisted Maneuvers

Depending on the level of autonomy of the UAV, a human pilot is involved to some extent in controlling the UAV. In general, the speed of the rotation modes determines a UAV’s responsiveness to maneuvering commands. If the rotation modes are unstable or lightly damped, it is typically difficult for a human pilot to manually control the UAV. Therefore, a stability augmentation system (SAS) is required to ensure that these modes have the desired dynamic characteristics. In addition to stabilizing the mode, a control augmentation system is intended in order to provide a specific response to the command. Even though slow flight modes such as phugoid and spiral can be manually controlled, an automatic control system is necessary to relieve the pilot of hand flying because it is undesirable for a pilot to pay constant attention. An autopilot is an automatic control system that provides both pilot relief and specialized functions such as path following and automatic landing [32].

**Figure 7:**
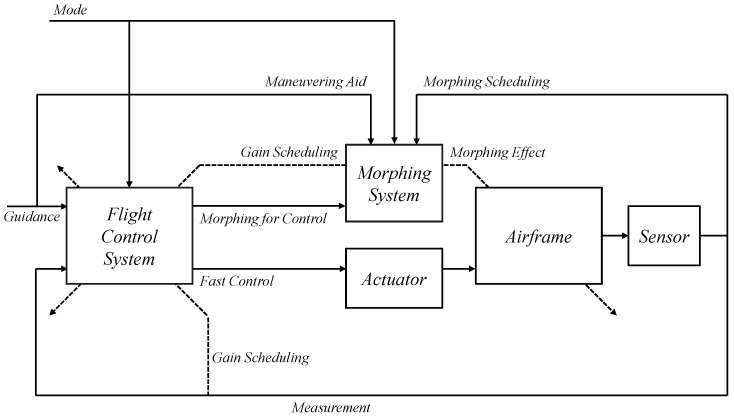
Control system design framework for morphing UAV.

In this study, the control system design framework shown in Figure 7 was considered. The flight control system (FCS) depends on the control mode, guidance commands, and scheduling parameters. The CAS modes and autopilot modes for the longitudinal and lateral-directional channels were considered for the control modes. The guidance command was determined according to the intended form of flight. The controller gains of the FCS were scheduled on the morphing parameters and the flight conditions in a manner that ensures stability, and the actuator commands were computed where the morphing actuator is also included in the control inputs. The morphing system governs the morphing configuration in a manner that satisfies the control command, aids the commanded maneuver, and provides the desired dynamic characteristics based on flight conditions. Conventional control surfaces are usually designed to provide aerodynamic forces and moments primarily for the intended channel with minimal cross-coupling effects and impacts on the mass properties. However, the morphing configuration change affects the characteristics of the airframe dynamics differently than the conventional control surfaces.

In this section, CASs are designed for morphing-assisted maneuvers of morphing UAVs based on LPV methods to provide a rigorous stability guarantee under arbitrary morphing. A longitudinal CAS is designed to track the normal acceleration command in Section 3.1. A lateral-directional CAS is designed to track the AOS and roll rate commands in Section 3.2. The CASs utilize the symmetric morphing parameter for an improved performance and the asymmetric morphing parameter for flight control. The symmetric morphing strategy is discussed in Section 3.3. In Section 3.4, numerical simulation is performed for the push-over and pull-up and the high-g turn to demonstrate the effectiveness of the proposed scheme. The proposed gain self-scheduled flight control system is compared with the gain-scheduled $H_{\infty }$ controller.

### 3.1. Longitudinal CAS Design for Normal Acceleration Control

The design of a normal acceleration CAS is based on LPV methods for controlling longitudinal motion. When the pilot must maneuver the UAV to its performance limits, high-performance UAVs require a specialized CAS. In this situation, normal acceleration is an appropriate variable for controlling the pitch axis. The accelerometer output has a component proportional to the AOA, allowing the short-period unstable mode to be stabilized. In addition, the accelerometer is typically less noisy and more reliable than the AOA sensor.

Note that the transfer function between the elevator deflection and normal acceleration has a nonminimum phase (NMP) zero. The initial response to a negative step elevator deflection will be a negative normal acceleration, followed by the expected positive normal acceleration. When the elevator is deflected upward to produce a positive normal acceleration, the tail experiences an increase in downward force. Consequently, the center of mass may momentarily fall, causing normal acceleration to decrease before increasing again. Therefore, the NMP behavior must be taken into account when designing the normal acceleration control system. The remaining degree of freedom in the throttle setting can be manually adjusted, or an autothrottle can be employed.

**Figure 8:**
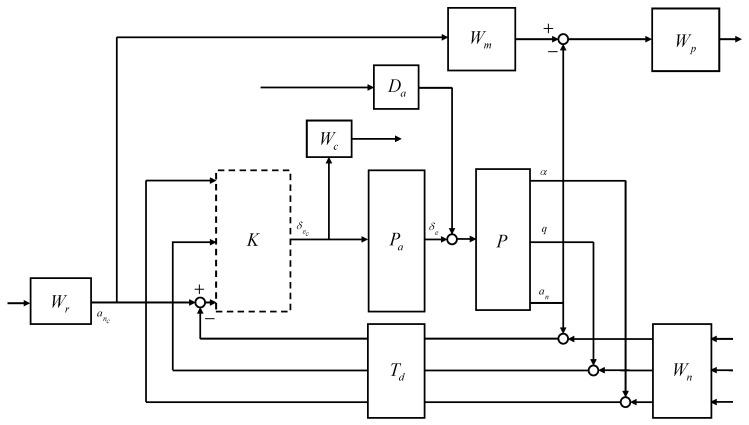
Closed-loop interconnection structure of the longitudinal CAS.

The closed-loop interconnection structure shown in Figure 8 was considered for the design of the longitudinal CAS. The 1-degree-of-freedom controller was considered. The open-loop plant *P* was composed of the state equation, Equation (50), and the output equation, Equation (51). The control design problem was cast in the model-matching framework. The control objective was to minimize the weighted normal acceleration model matching error and the weighted control effort against the command, noise, and disturbance.

The external input vector *w* was assumed to be a broadband $L_{2}$ signal. For design convenience, the $L_{2}$-norm of the external inputs can be regarded as unity. In this case, the control objective becomes making the performance output less than unity, and the weighting filters were designed accordingly. Note that the frequency-dependent weights can be designed as real, rational, and proper transfer matrices whose elements may depend on the scheduling parameter; that is, the performance objectives can also be scheduled on the scheduling parameter. The open-loop plant *P* was the LPV model obtained in Section 2.2. The actuator model $P_{a}$ was designed to reflect the realistic responses of the control surfaces to the control input commands. The reference weight $W_{r}$ corresponds to the maximum expected guidance command. The model weight $W_{m}$ corresponds to the ideal model response to the unit step guidance command. The performance weight $W_{p}$ shapes the relative importance of the model matching error throughout the frequency range. The noise weight $W_{n}$ corresponds to the expected sensor noise level across the frequency range. The control weight $W_{c}$ shapes the control effort penalty to the actuator command in the undesirable frequency range. The disturbance weight $D_{a}$ corresponds to the expected disturbance to the actuator output. The time delay $T_{d}$ corresponds to the expected transport delay.

The elevator was modeled as a first-order system with a bandwidth of 5 Hz. Except for some highly agile maneuvers, in most cases, the vertical acceleration command does not exceed 2 g, and, therefore, the reference weight was set to 2 g. The ideal model for the normal acceleration response to the command was set to have a natural frequency of 2.5 rad/s and a damping ratio of 0.8 to allow for some overshoot. Performance weights were set to ensure the model matching error does not exceed five times the normal acceleration measurement noise level at low frequencies. The sensor measurement error was modeled as white Gaussian noise, and the standard deviation was set to match the sensor specifications of the GTM. In the elevator model, a disturbance of 0.01 deg was added below 0.5 rad/s to account for errors due to the dead zone, saturation, and rate limit. A first-order Padé approximant corresponding to a transport delay of 30 ms was applied to all sensor measurements. The Laplace transform of a time delay of T is $e^{-sT}$, and the exponential transfer function can be approximated by a rational transfer function using Padé approximation formulas. Given the order of a rational function, the Padé approximant is known as the best approximation. High-order Padé approximations produce transfer functions with clustered poles. In general, Padé approximations with high order (N>10) are not preferred because their poles are sensitive to perturbations.

The generalized open-loop plant has 11 states and depends on three scheduling parameters. The LPV controller was synthesized to satisfy performance objectives according to the performance specifications defined in the generalized open-loop plant. The synthesized LPV controller guarantees that the closed-loop system is quadratically stable and that the $L_{2}$ gain from the external input $w\in L_{2}$ to the performance output z is less than γ≥0 under arbitrary time-varying scheduling parameter ρ∈P. The LPV synthesis problem was solved twice. In the first iteration, an optimal solution that minimizes the induced $L_{2}$-norm of $F_{l}\left(G,K\right)$ was obtained. In the second iteration, there is a suboptimal solution whose γ is at most 20% greater than the γ obtained in the first iteration for better numerical conditioning. As a result of LPV synthesis, suboptimal γ=2.1505 was obtained, which is 10.9% greater than the optimal γ=1.9387. The $H_{\infty }$ controller was synthesized with respect to the pointwise LTI models, whose γ ranges from 0.2006 to 0.2258. Note that the $H_{2}$ controller can also be synthesized in this way. The synthesis time is largest in the LPV controller and smallest in the $H_{2}$ controller.

### 3.2. Lateral-Directional CAS Design for Turn Coordination and Roll Rate Control

Regarding lateral-directional control, the most prevalent control augmentation system is the roll-rate command system. This mechanism can be constructed to roll the aircraft about its own velocity vector rather than the body axis. The roll-yaw stability augmentation system is adequate for the majority of aircraft, but a more refined lateral-directional CAS is required for aircraft that must maneuver rapidly at a high angle of attack (AOA). At high alpha, the lateral aerodynamic control surfaces have a tendency to cause the aircraft to roll about its longitudinal axis, which can lead to extremely undesirable phenomena such as the kinematic coupling of alpha and beta. The primary purpose of a roll is to initiate a turn, which is accomplished by utilizing the AOA to generate the lift that will ultimately produce the required centripetal acceleration.

**Figure 9:**
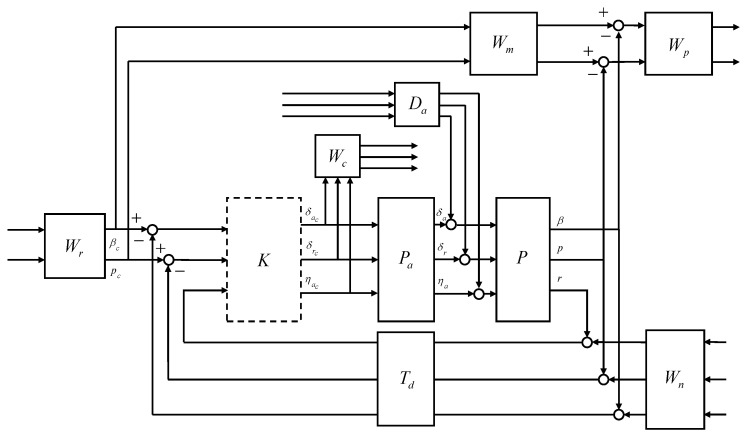
Closed-loop interconnection of the lateral-directional CAS.

The interconnection shown in Figure 9 was considered for the control design. The aileron, rudder, and morphing actuator were modeled as first-order systems, where the bandwidth was set to 5 Hz for the conventional control surfaces and 0.5 Hz for the morphing actuator to address a relatively slow response of the morphing actuators. Except for some highly agile maneuvers, in most cases, the AOS and the roll rate command do not exceed 3 deg and 10 deg/s, respectively. The ideal model for the AOS response to the command was set to have a natural frequency of 2 rad/s and a damping ratio of 0.8 to allow for some overshoot. The ideal model for the roll rate response to the command was set to have a natural frequency of 10 rad/s and a damping ratio of 0.8 to allow for faster convergence. Performance weights were set to ensure that the model matching error does not exceed two times the corresponding measurement noise level at low frequencies. The sensor measurement error was modeled as white Gaussian noise, and the standard deviation was set to match the sensor specifications of the GTM. In the actuator models, a disturbance of 0.01 deg was added below 0.5 rad/s to account for errors due to the dead zone, saturation, rate limit, and additional uncertainties arising from complex morphing mechanisms. A first-order Padé approximant corresponding to a transport delay of 30 ms was applied to all sensor measurements. The LPV controller and the $H_{\infty }$ controller were synthesized.

### 3.3. Span Morphing Strategy

#### 3.3.1. Effects of Span Morphing

In aircraft design, the wing span is one of the most crucial geometrical elements. It is directly related to the lift-induced drag of the wing: as the span increases, an increasing proportion of the inboard wing behaves as if in two-dimensional flow conditions [5]. Due to the increased distance between the vortices and the wing sections at the root, the downwash caused by the wingtip vortices is reduced in the inboard region of the wing. If the wing is swept, significant variations in the CM and CP can be anticipated, altering the trim and stability conditions. With span morphing, both the wing span and lifting area are altered. Therefore, the wing’s AR, which affects the induced drag, and the wetted area, which affects the profile drag, are both varied. For increasing $C_{L}$ values, configurations with a larger span may offer not only more lift but also less drag due to lower AOA requirements and less induced drag. Consequently, wing span primarily influences the total wing drag due to its effect on induced drag. To maximize the lift-to-drag ratio of the wing throughout the flight envelope, one can anticipate greater aerodynamic gains when operating at high $C_{L}$ values, i.e., at slower flight speeds.

Additionally, the effect on maneuverability must be taken into account. An increase in span has further effects on stability and control. The first and most obvious result is an increase in rolling MOI. Because the rolling MOI is proportional to the square of the distance between the mass elements and the longitudinal axis of the aircraft, there is a quadratic rise with the span. Therefore, if it is not possible to reduce the wing’s structural mass toward the tips, even if the device generating the rolling moment stays close to the wingtip, large reductions in the achievable roll rate can be anticipated. Increased the aerodynamic damping of the rolling motion due to a higher wingtip speed also contributes to a loss in maneuverability, which increases rolling stability. If the wing is swept, an increase in the span is likely to induce a change in the aircraft’s center of gravity, which will become more noticeable as the wing structural mass grows in relation to the aircraft’s mass. The position of the wing’s aerodynamic center is also modified. In turn, the trim drag and static stability margin are affected by changes in the center of gravity (CG) and aerodynamic center (AC). Similarly, if the span is raised asymmetrically, the CG and AC will move off the symmetry plane of the airplane, and there will be a stronger coupling between the longitudinal and lateral movements as the aircraft becomes more asymmetric [5].

#### 3.3.2. Criteria for Span Variation

For improved agility and maneuverability, the symmetric morphing parameter should be increased when the normal acceleration command is large, and the symmetric morphing parameter should be decreased when the roll rate command is large.

### 3.4. Nonlinear Simulation of Morphing-Assisted Maneuvers

The GTM program was used to simulate a morphing aircraft’s flight dynamics. It implements general motion equations for rigid bodies for the vehicle dynamics and derives aerodynamic forces using a standard coefficient expansion performed as table lookups. The dynamics of the servo actuator and sensor bandwidth and errors were also included.

#### 3.4.1. Push-Over and Pull-Up

**Figure 10:**
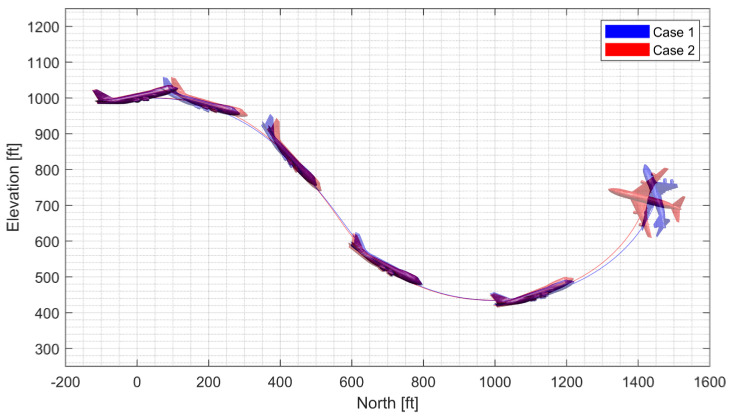
Flight trajectory of the push-over and pull-up (LPV).

**Figure 11:**
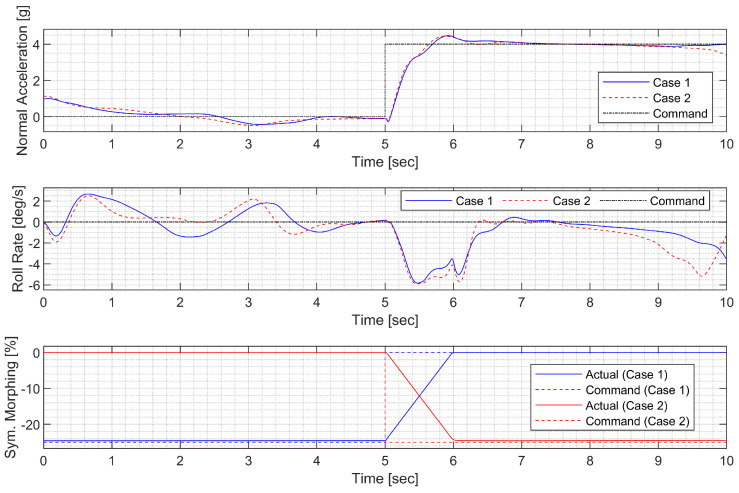
Controlled states and symmetric morphing parameter history for the push-over and pull-up (LPV).

**Figure 12:**
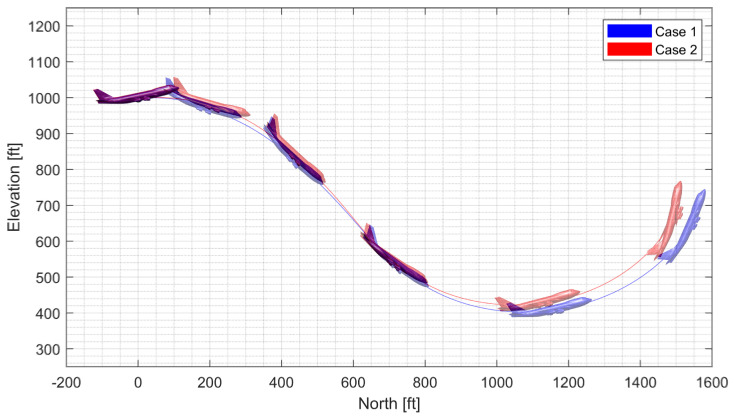
Flight trajectory of the push-over and pull-up ($H_{\infty }$).

**Figure 13:**
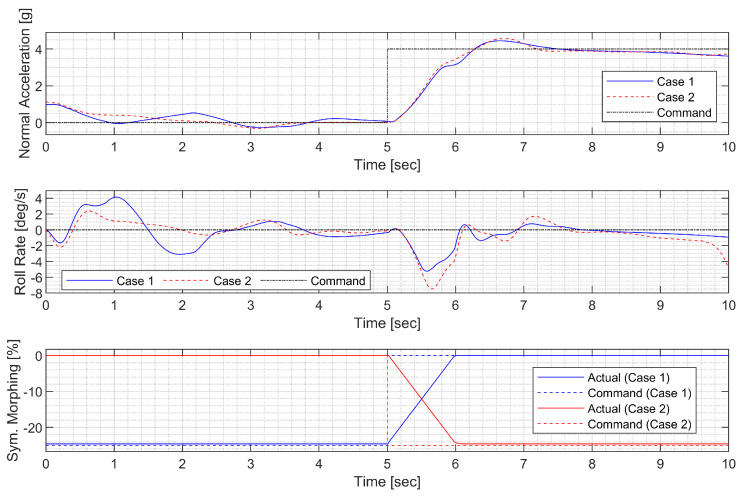
Controlled states and symmetric morphing parameter history for the push-over and pull-up ($H_{\infty }$).

First, a push-over and pull-up maneuver was considered. A normal acceleration command of 0g is engaged for the first 5 s; then, the 4g command is engaged. The throttle is fixed to 90% throughout the flight. Figure 10 and Figure 11 show the result using the LPV controller, and Figure 12 and Figure 13 show the result using the $H_{\infty }$ controller. In case 1, the aircraft performs a push-over with the span shortened and then pulls up with the span extended, and, in case 2, the span is controlled in the opposite way. Both aircraft perform well before entering the stall, but the configuration profile in case 1 is superior in maintaining a longer pull-up. Additionally, the normal acceleration tracking performance of the $H_{\infty }$ controller degrades when the morphing configuration changes rapidly, while the LPV controller shows a faster convergence to the command. However, the LPV-based CAS results in a larger roll angle because the CAS tries to control only the roll rate instead of the roll angle, which can be improved by implementing a roll angle autopilot. The simulation results for the $H_{2}$ controller are omitted because the results are very similar to those for the $H_{\infty }$ controller.

#### 3.4.2. High-G Turn

**Figure 14:**
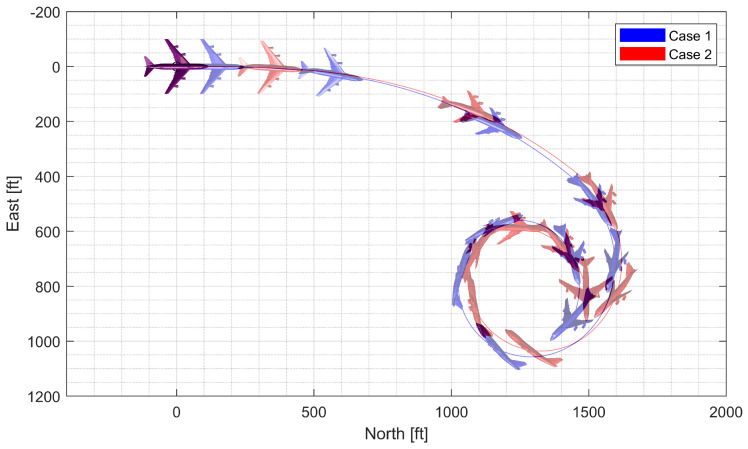
Flight trajectory of the high-g turn (LPV).

**Figure 15:**
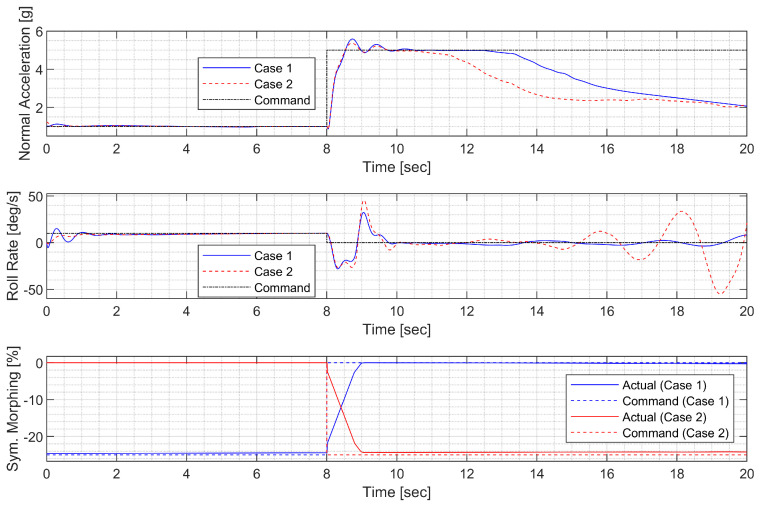
Controlled states and symmetric morphing parameter history for the high-g turn (LPV).

**Figure 16:**
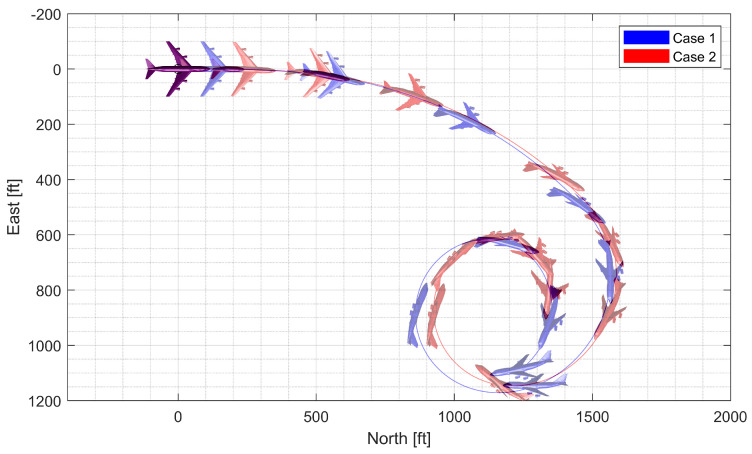
Flight trajectory of the high-g turn ($H_{\infty }$).

**Figure 17:**
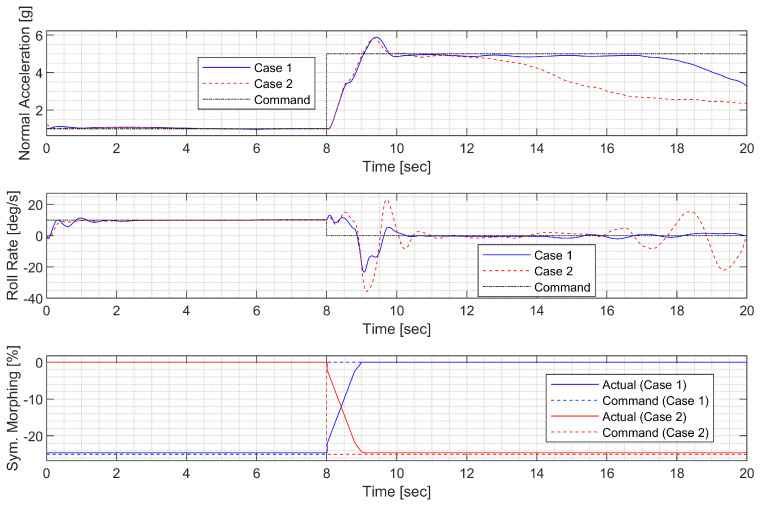
Controlled states and symmetric morphing parameter history for the high-g turn ($H_{\infty }$).

Next, a high-g turn was considered. A roll rate command of 10 deg/s is engaged for the first 8 s to reach an 80 deg roll angle; then, a 0 deg/s command is engaged. A normal acceleration command of 1g is engaged for the first 8 s; then, the 5 g command is engaged. The throttle is fixed to the maximum throughout the flight. In case 1, the aircraft rotates on the roll axis with the span shortened, then increases the span and increases the normal acceleration to make a high-g turn. In case 2, the span is controlled in the opposite way. Figure 14, Figure 15, Figure 16 and Figure 17 shows that both aircraft perform well before entering the stall, but one configuration profile in case 1 is superior in maintaining a longer turn. Note that the LPV controller is quicker in convergence, but the $H_{\infty }$ controller is better at maintaining the command longer where the configuration is not changed.

## 4. Autopilot Design Based on LPV Methods for Morphing-Assisted Flights

When the flight management system, including autopilots, is activated, a UAV can complete an entire mission without manual remote control. The majority of flying quality requirements have no direct bearing on the autopilot design. Pilot relief autopilot modes require the autopilot to meet standards for steady-state error and disturbance rejection while placing less importance on the dynamic response. To prevent distracting or potentially hazardous transient motions, careful consideration must also be given to how the autopilot is turned on and off.

In this section, an autopilot is designed for the morphing-assisted flight of morphing UAVs based on LPV methods. A longitudinal autopilot was designed to track the true airspeed and altitude commands. A lateral-directional autopilot was designed to track the AOS and roll angle commands. The autopilot utilizes symmetric and asymmetric morphing for improved agility and maneuverability, respectively. Numerical simulation is performed to demonstrate the effectiveness of the proposed scheme.

### 4.1. Longitudinal Autopilot Design for Airspeed and Altitude Control

An airspeed and altitude autopilot was designed to control longitudinal motion. Altitude hold is a vital pilot relief option that allows an aircraft to be maintained at a predetermined altitude in accordance with air traffic control requirements in an air route corridor. Typically, the speed-hold autopilot is utilized during climb and descent. During a climb, the throttles may be set to a relatively high level of power, and the elevator will receive a speed input to maintain a constant speed. While the speed will vary based on altitude, keeping the speed constant will maximize fuel efficiency. In addition, the descent will be conducted at a constant speed, with the throttles set to near idle. For effective cruising, both the throttle and the elevator will be used to maintain speed and altitude at the cruising altitude.

**Figure 18:**
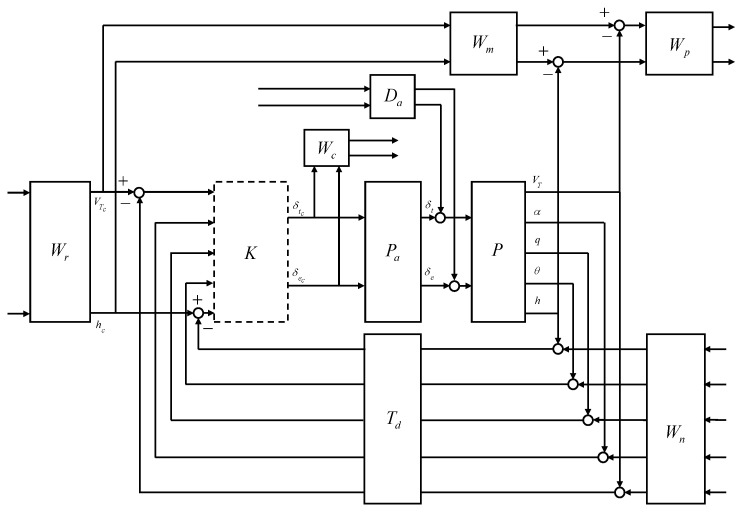
Closed-loop interconnection of the longitudinal autopilot.

In longitudinal aircraft dynamics, there is significant coupling between the two control inputs (engine throttle and elevator deflection angle) and the two main outputs (speed and altitude). The interconnection shown in Figure 18 was considered for the control design.

The engine lag was modeled as a second-order system with NMP zero. The reference weight was set to 4 kt for the true airspeed and 10 ft for the altitude. The ideal models for the true airspeed and altitude response to the command were set to have a natural frequency of 2 rad/s and a damping ratio of 0.8 for a gentle response. Performance weights were set to ensure that the model matching error does not exceed two times the measurement noise levels at low frequencies. The sensor measurement error was modeled as white Gaussian noise, and the standard deviation was set to match the sensor specifications of the GTM. In the actuator model, a disturbance of 0.01 deg was added below 0.5 rad/s to account for various errors.

### 4.2. Lateral-Directional Autopilot Design for Turn Coordination and Roll Angle Control

A roll angle autopilot was designed based on LPV methods to control lateral-directional motion. The purpose of the autopilot is to keep the wings level. To achieve a coordinated turning motion, additional control systems must be used to regulate the sideslip and pitch rate if the aircraft is maintained at an angle other than at the wings level. In turn, the pitch rate command will determine whether the aircraft gains or loses altitude. If the roll reference can be altered, the aircraft can be directed in any direction with single control. These control systems can provide inner loops for various autopilots, allowing an aircraft to maintain a constant compass heading or follow a radio navigation beam despite crosswinds.

**Figure 19:**
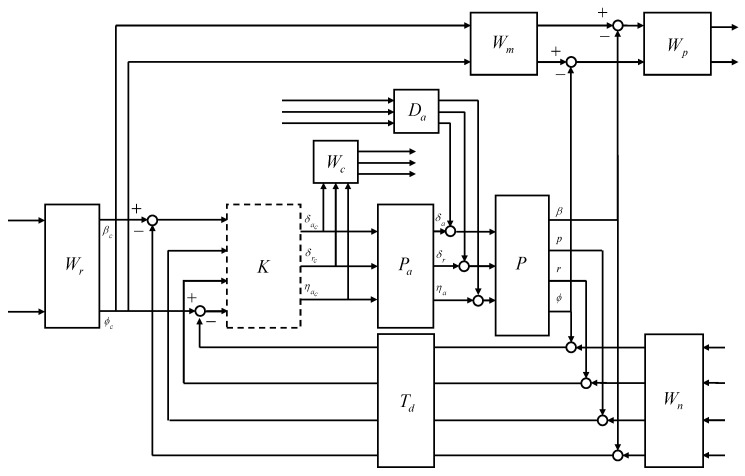
Closed-loop interconnection of the lateral-directional autopilot.

There is significant coupling between the control inputs and the main outputs in the lateral-direction aircraft dynamics. The interconnection shown in Figure 19 was considered for the control design.

The aileron, rudder, and morphing actuator were modeled in the same way as in the CAS design. The reference weight was set to 30 deg for the roll angle command, and this condition can be forced in the guidance command. The ideal model for the AOS response to the command was set to have a natural frequency of 2 rad/s and a damping ratio of 0.8 to allow for some overshoot. The ideal model for the roll rate response to the command was set to have a natural frequency of 5 rad/s and a damping ratio of 0.8 to allow for faster convergence. Performance weights were set to ensure that the model matching error does not exceed two times the corresponding measurement noise level at low frequencies. The sensor measurement error was modeled as white Gaussian noise, and the standard deviation was set to match the sensor specifications of the GTM. In the actuator model, a disturbance of 0.01 deg was added below 0.5 rad/s to account for errors due to the dead zone, saturation, rate limit, and additional uncertainties arising from complex morphing mechanisms.

### 4.3. Nonlinear Guidance for Trajectory Tracking

A lookahead distance was used to compute the desired course angle. The lateral acceleration command was generated from the guidance law [36].

(55) $$a_{s_{cmd}}=2\frac{V^{2}}{d}\sin\sigma$$

To convert the lateral acceleration command into the bank angle command, the relation $a_{s}\approx g\phi$ derived from the aircraft coordinate turn was utilized. A heading controller was used to compute the required roll angle.

(56) $$\phi_{c}=\tan^{-1}\left(K_{\psi }\frac{V}{g}\left(\psi_{c}-\psi \right)\right)$$

(57) $$\dot{\psi }+K_{\psi }\psi =K_{\psi }\psi_{c}$$

(58) $$\frac{\psi (s)}{\psi_{c}\left(s\right)}=\frac{K_{\psi }}{s+K_{\psi }}$$

The heading controller gain was set to $K_{\psi }=3.9$.

The optimal span morphing parameter can be determined as a function of flight conditions (airspeed and altitude). Furthermore, the span morphing parameter can be increased to attenuate the altitude drop when a large roll angle command is engaged. In this study, the span morphing parameter deviation from the setpoint was obtained in proportion to the roll angle command.

### 4.4. Nonlinear Simulation of Morphing-Assisted Flights

#### 4.4.1. Waypoint Following at Low Altitude

**Figure 20:**
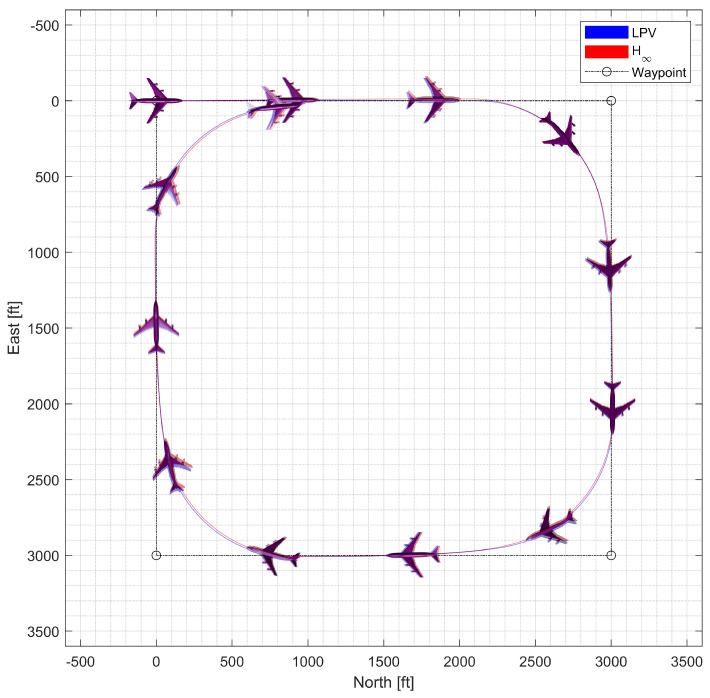
Flight trajectory of the waypoint-following flight.

**Figure 21:**
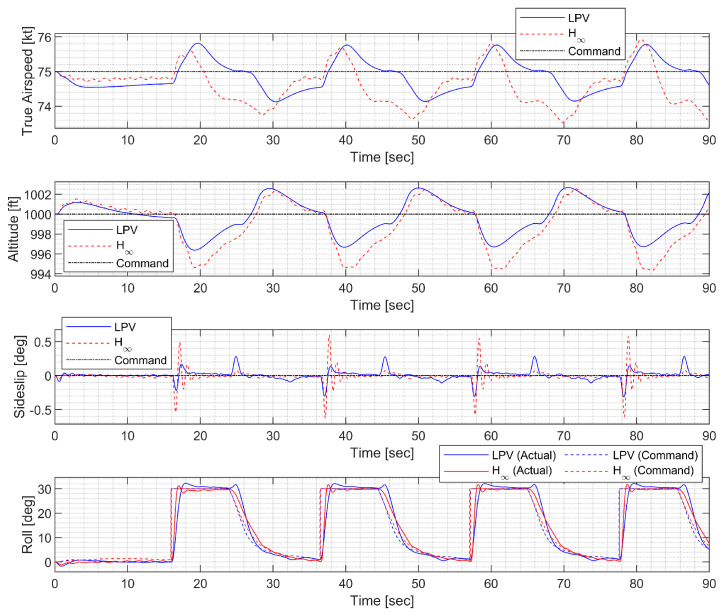
State history of the waypoint-following flight.

**Figure 22:**
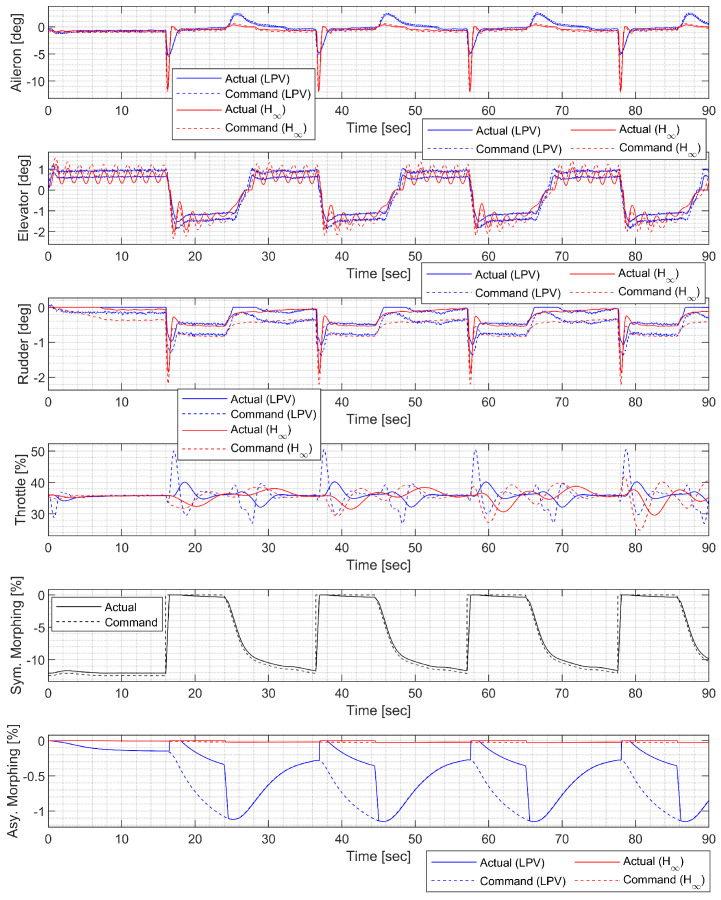
Input history of the waypoint-following flight.

In the waypoint following at low altitude, the morphing UAV was commanded to follow four waypoints placed 3000 ft apart at the same altitude. The resulting trajectory and state response are shown in Figure 20, Figure 21 and Figure 22. Notably, the $H_{\infty }$ controller exhibits larger oscillation in both the longitudinal and lateral-directional motions, possibly due to rapid parameter variations.

#### 4.4.2. Circular Trajectory Tracking at High Altitude

**Figure 23:**
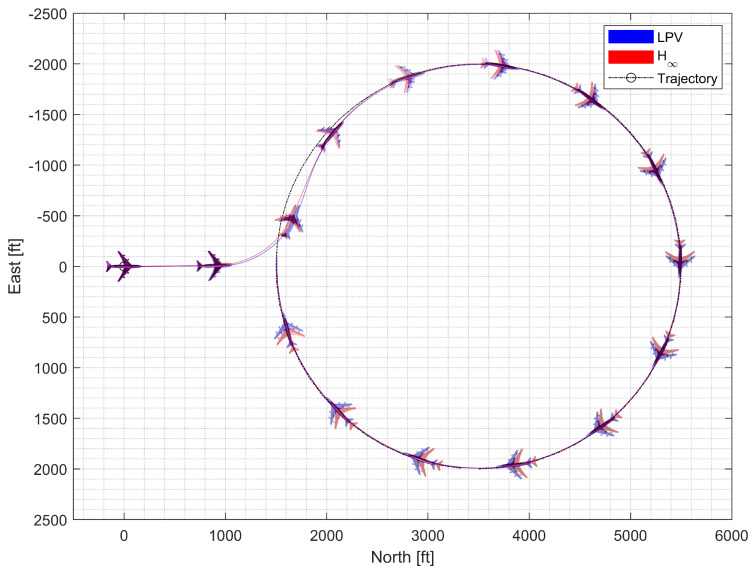
Flight trajectory of the circular trajectory-following flight.

**Figure 24:**
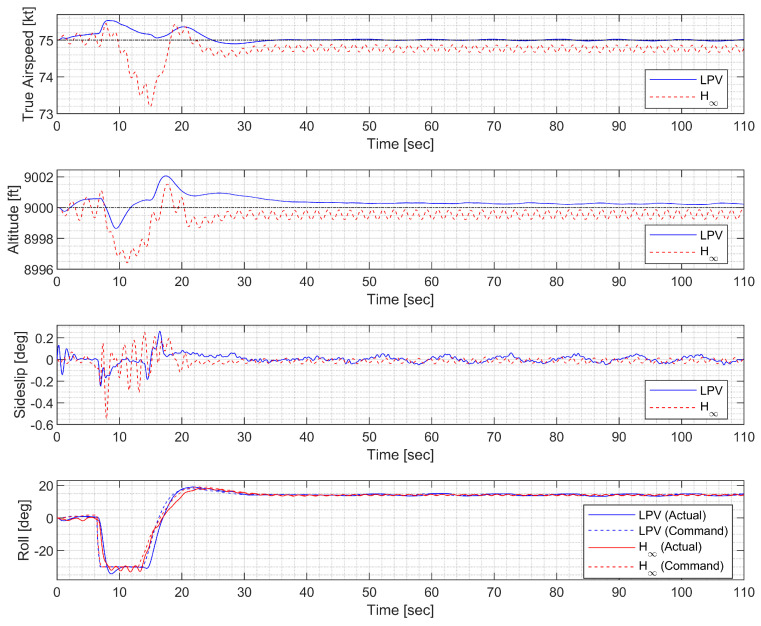
State history of the circular trajectory-following flight.

**Figure 25:**
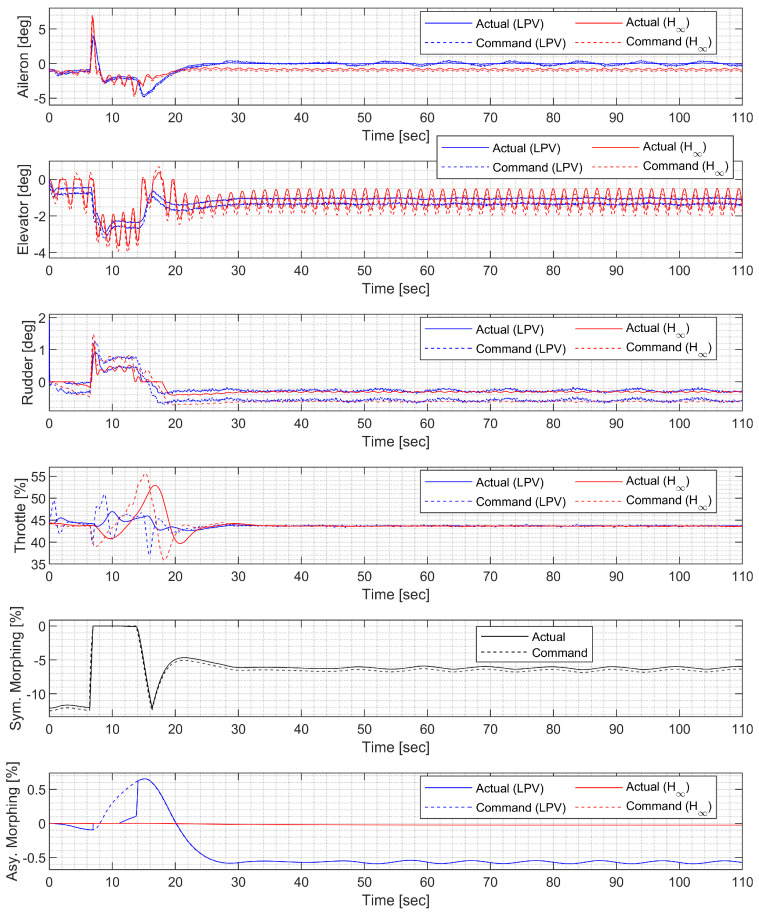
Input history of the circular trajectory-following flight.

In the circular trajectory tracking at high altitude, the morphing UAV was commanded to follow a horizontal orbit with a radius of 2000 ft. The resulting trajectory and state response are shown in Figure 23, Figure 24 and Figure 25. The $H_{\infty }$ controller exhibits undesirable chattering phenomena in both the longitudinal and lateral-directional motions as a result of aggressive high gains.

#### 4.4.3. Helical Ascent under Fast Morphing

**Figure 26:**
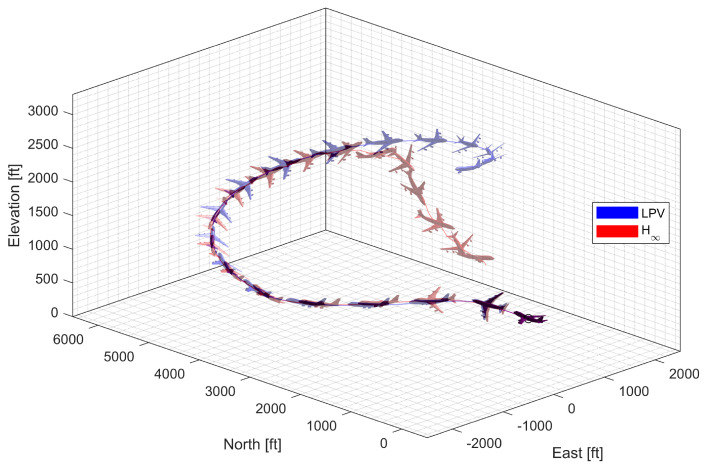
Flight trajectory of the helical ascent.

**Figure 27:**
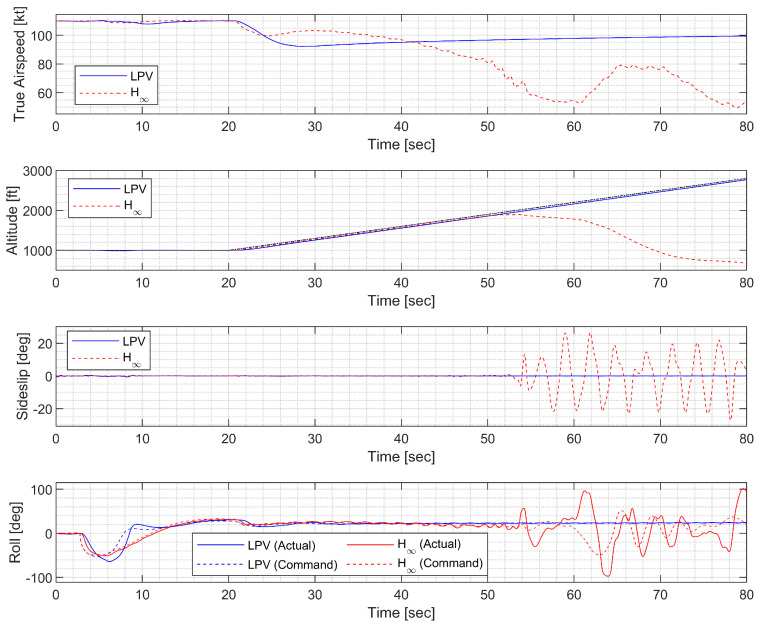
State history of the helical ascent.

**Figure 28:**
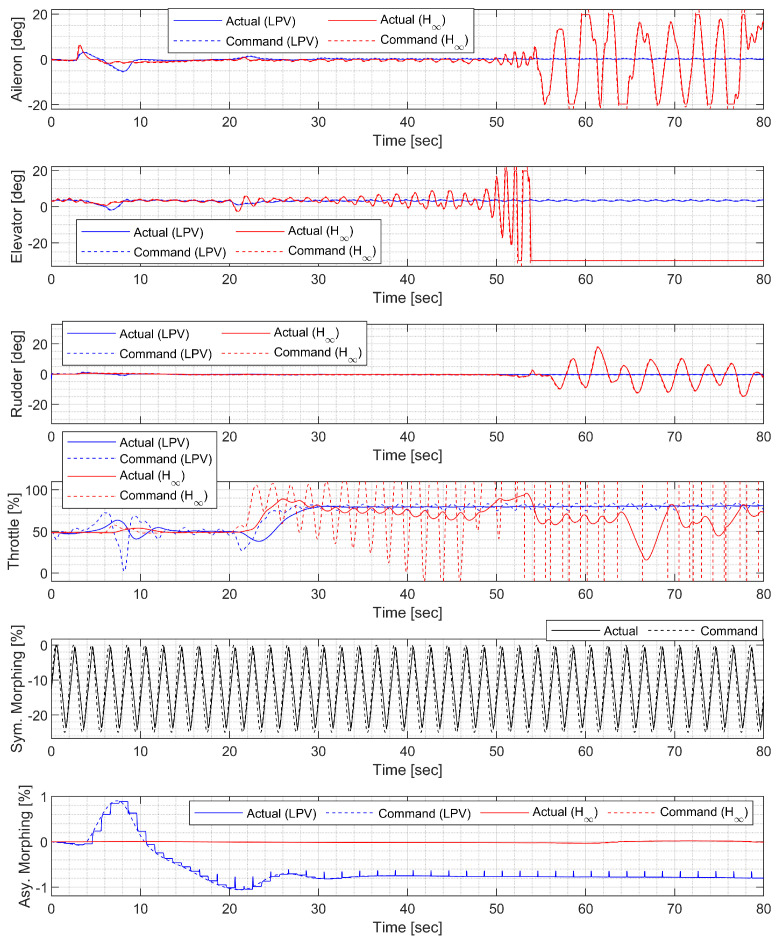
Input history of the helical ascent.

In the helical ascent under fast morphing, the morphing UAV was commanded to follow a helical path with a radius of 2000 ft and a climb rate of 30 ft/s, which corresponds to a flight path angle of approximately 9.18 deg. The resulting trajectory and state response are shown in Figure 26, Figure 27 and Figure 28. Extremely fast morphing was used to test the marginal performance of the designed controllers. Note that the oscillation of the $H_{\infty }$ controller gradually grows and eventually diverges, whereas the LPV controller maintains the tracking performance.

#### 4.4.4. Spiral Descent with Morphing Scheduling

**Figure 29:**
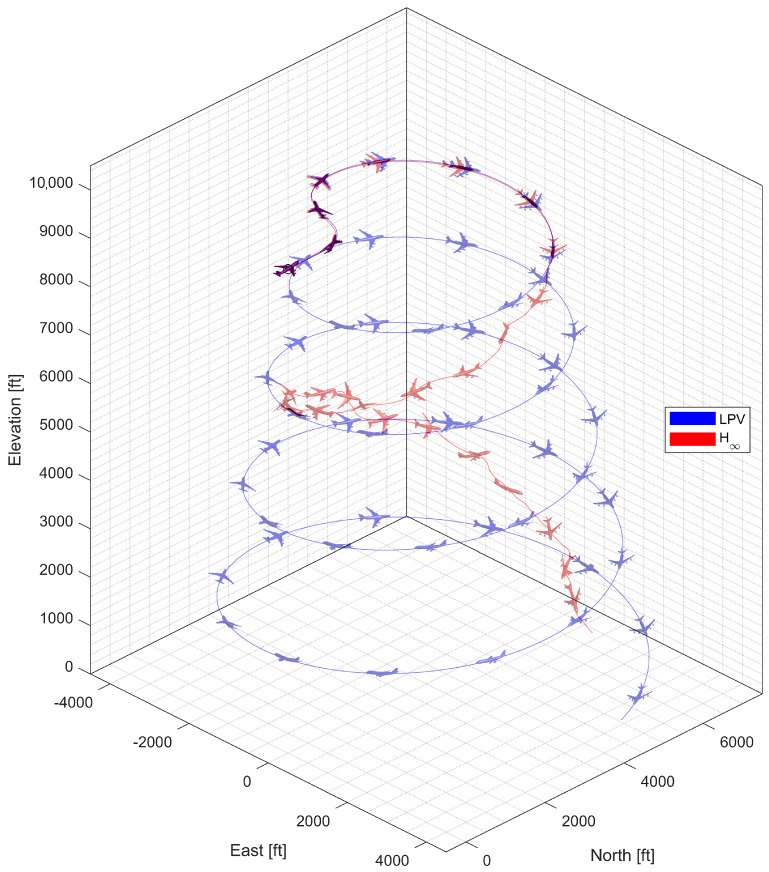
Flight trajectory of the spiral descent.

**Figure 30:**
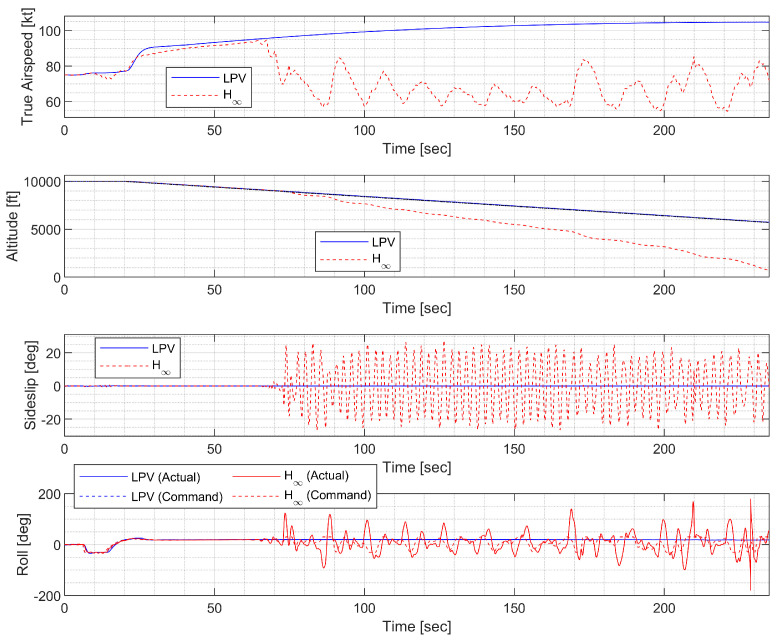
Statehistory of the spiral descent.

**Figure 31:**
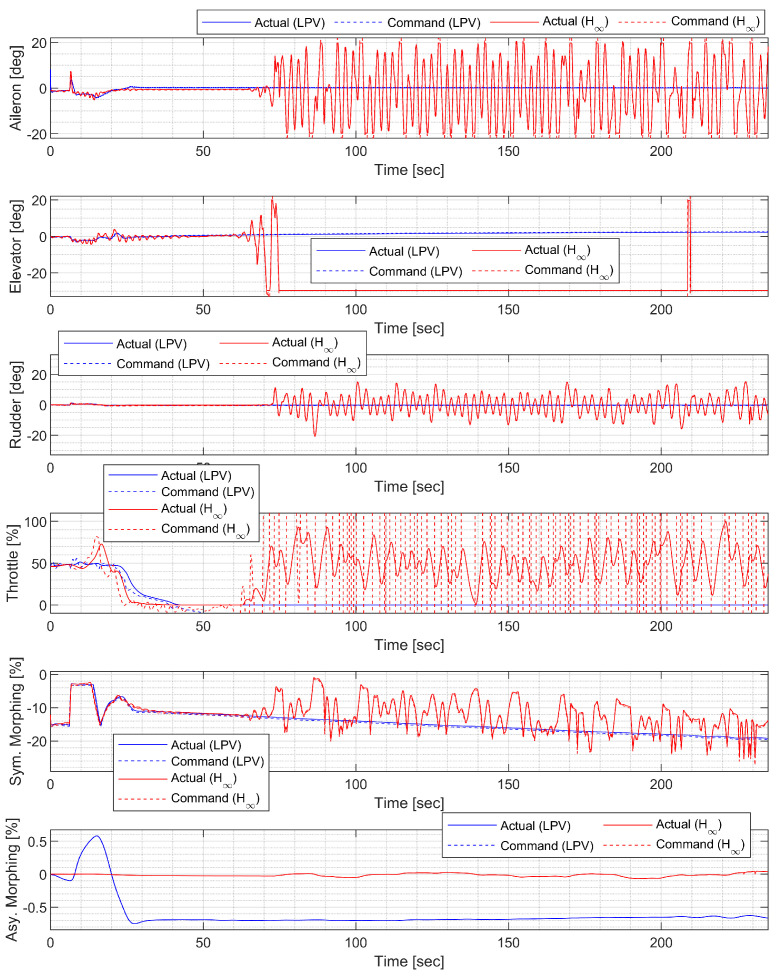
Input history of the spiral descent.

In spiral descent with morphing scheduling, the morphing UAV was commanded to follow a helical path with a radius of 2000 ft and a rate of descent of −20 ft/s, which corresponds to an approximate flight path angle of −8.98 deg. The symmetric morphing configuration was scheduled on the flight conditions so that the aerodynamic performance is improved. The resulting trajectory and state response are shown in Figure 29, Figure 30 and Figure 31. Note that the oscillation of the $H_{\infty }$ controller quickly loses stability even when the scheduling parameter undergoes gentle changes, whereas the LPV controller maintains the tracking performance.

## 5. Conclusions

A novel framework is proposed for the flight control of morphing unmanned aerial vehicles (UAVs). The proposed scheme reflects the benefits of symmetric span morphing in the longitudinal performance while utilizing asymmetric span morphing for roll control. The control system was designed based on linear parameter-varying (LPV) methods that naturally suit the parameter-varying nature of morphing UAVs. First, a high-fidelity model of an asymmetric variable-span morphing UAV was derived from the NASA generic transport model. Second, the control augmentation system was designed based on LPV methods to track normal acceleration and roll rate commands while maintaining a small angle of sideslip. Finally, the autopilot was designed based on LPV methods to track airspeed, altitude, and roll angle commands while maintaining a small angle of sideslip.

**Remark** **4.** *The presented LPV controller was obtained by solving complex optimization problems in the design process. However, once the design was carried out, the amount of online calculation was very small because the resulting controller has the form of a gain-scheduled linear controller. Therefore, the amount of online calculation is not a problem in practice.*

Future research could focus on a technique for determining the optimal shape in real time. The optimal shape can also be determined offline by considering flight conditions only. Therefore, the online optimum shape determiner must be able to appropriately consider both current flight conditions and external guidance commands. When a large amount of lift is needed, for instance, a longer span can be extended even under the same flight conditions. When a high-speed dash or rapid rotation of a roll axis is needed, however, a short span can be advantageous. When only one morphing parameter is considered as a scheduling parameter, the problem can be easily solved using a straightforward line search. However, with two or more morphing parameters, it may be more challenging to solve the problem.

## Data Availability

Not applicable.

## References

[1] Cao B., Li M., Liu X., Zhao J., Cao W., Lv Z. (2021). Many-Objective Deployment Optimization for a Drone-Assisted Camera Network. IEEE Trans. Netw. Sci. Eng..

[2] Lv Z., Qiao L., Hossain M.S., Choi B.J. (2021). Analysis of Using Blockchain to Protect the Privacy of Drone Big Data. IEEE Netw..

[3] Valasek J. (2012). Morphing Aerospace Vehicles and Structures.

[4] Wickenheiser A.M., Garcia E. (2007). Aerodynamic modeling of morphing wings using an extended lifting-line analysis. J. Aircr..

[5] Dimino I., Lecce L., Pecora R. (2017). Morphing Wing Technologies: Large Commercial Aircraft and Civil Helicopters.

[6] Barbarino S., Bilgen O., Ajaj R.M., Friswell M.I., Inman D.J. (2011). A review of morphing aircraft. J. Intell. Mater. Syst. Struct..

[7] Shao P., Wu J., Wu C., Ma S. (2019). Model and robust gain-scheduled PID control of a bio-inspired morphing UAV based on LPV method. Asian J. Control.

[8] Jiang W., Wu K., Wang Z., Wang Y. (2020). Gain-scheduled control for morphing aircraft via switching polytopic linear parameter-varying systems. Aerosp. Sci. Technol..

[9] Xu W., Li Y., Lv M., Pei B. (2022). Modeling and switching adaptive control for nonlinear morphing aircraft considering actuator dynamics. Aerosp. Sci. Technol..

[10] Xu D., Hui Z., Liu Y., Chen G. (2019). Morphing control of a new bionic morphing UAV with deep reinforcement learning. Aerosp. Sci. Technol..

[11] Jiao X., Jiang J. (2015). Learning control law of mode switching for hypersonic morphing aircraft based on type-2 TSK fuzzy neural network. Int. J. Mach. Learn. Comput..

[12] Valasek J., Doebbler J., Tandale M.D., Meade A.J. (2008). Improved adaptive-reinforcement learning control for morphing unmanned air vehicles. IEEE Trans. Syst. Man Cybern. Part B Cybern..

[13] Wu Z., Lu J., Rajput J., Shi J., Ma W. (2015). Adaptive neural control based on high order integral chained differentiator for morphing aircraft. Math. Probl. Eng..

[14] Valasek J., Tandale M.D., Rong J. (2005). A reinforcement learning - Adaptive control architecture for morphing. J. Aerosp. Comput. Inf. Commun..

[15] Yao L., You S., Xiaodong L., Bo G. (2020). Control allocation for a class of morphing aircraft with integer constraints based on Levy flight. J. Syst. Eng. Electron..

[16] Liu J., Wang J. (2022). Incremental sliding-mode control and allocation for morphing-wing aircraft fast manoeuvring. Aerosp. Sci. Technol..

[17] Wen N., Liu Z., Sun Y., Zhu L. (2017). Design of LPV-based sliding mode controller with finite time convergence for a morphing aircraft. Int. J. Aerosp. Eng..

[18] Qiao F., Guo L., Shi J. (2020). Morphing aircraft flight control using nonlinear dynamic inversion. Advances in Guidance, Navigation and Control—Lecture Notes in Electrical Engineering.

[19] Liang X., Wang Q., Xu B., Dong C. (2021). Back-stepping Fault-tolerant Control for Morphing Aircraft Based on Fixed-time Observer. Int. J. Control. Autom. Syst..

[20] Wu K.J., Zhang P.X., Wu H. (2019). A new control design for a morphing UAV based on disturbance observer and command filtered backstepping techniques. Sci. China Technol. Sci..

[21] Gong L., Wang Q., Dong C. (2019). Disturbance rejection control of morphing aircraft based on switched nonlinear systems. Nonlinear Dyn..

[22] Li Y., Liu J., Shan J., Wang C., Chen Y. Disturbance Rejection LPV Control of Morphing Aircraft Based on Extended State Observer. Proceedings of the 2022 International Conference on Guidance, Navigation and Control.

[23] Yue T., Wang L., Ai J. (2013). Gain self-scheduled H_∞_ control for morphing aircraft in the wing transition process based on an LPV model. Chin. J. Aeronaut..

[24] Lu B., Wu F. (2006). Probabilistic robust linear parameter-varying control of an F-16 aircraft. J. Guid. Control. Dyn..

[25] Shin J.Y., Balas G.J., Kaya M.A. (2002). Blending methodology of linear parameter varying control synthesis of F-16 aircraft system. J. Guid. Control. Dyn..

[26] Lu B., Wu F., Kim S.W. (2006). Switching LPV control of an F-16 aircraft via controller state reset. IEEE Trans. Control Syst. Technol..

[27] Wang Z., Hou M., Hao M. Morphing-Aided Maneuver Control of Morphing Aircraft with Variable Wing Span and Sweep Angle. Proceedings of the 2022 International Conference on Guidance, Navigation and Control.

[28] Yao Z., Wu S. (2019). Intermittent gliding flight control design and verification of a morphing unmanned aerial vehicle. IEEE Access.

[29] Liu Y., Ban X., Wu F., Lam H.K. (2016). A gain-scheduling control approach for Takagi-Sugeno fuzzy systems based on linear parameter-varying control theory. J. Dyn. Syst. Meas. Control. Trans. ASME.

[30] Sabzalian M.H., Mohammadzadeh A., Lin S., Zhang W. (2020). A robust control of a class of induction motors using rough type-2 fuzzy neural networks. Soft Comput..

[31] Cunningham K., Cox D.E., Murri D.G., Riddick S.E. A piloted evaluation of damage accommodating flight control using a remotely piloted vehicle. Proceedings of the AIAA Guidance, Navigation, and Control Conference.

[32] Stevens B.L., Johnson E.N., Lewis F.L. (2015). Aircraft Control and Simulation: Dynamics, Controls Design, and Autonomous Systems.

[33] Bae J.S., Seigler T.M., Inman D.J. (2005). Aerodynamic and static aeroelastic characteristics of a variable-span morphing wing. J. Aircr..

[34] Anderson J.D. (1999). Aircraft Performance and Design.

[35] Ganguli S., Marcos A., Balas G. Reconfigurable LPV control design for Boeing 747-100/200 longitudinal axis. Proceedings of the American Control Conference.

[36] Park S., Deyst J., How J.P. A new nonlinear guidance logic for trajectory tracking. Proceedings of the AIAA Guidance, Navigation, and Control Conference and Exhibit.

